# Supramolecular Stabilization of Single-Molecule SERS: Cucurbit[7]uril Encapsulation of Thionine

**DOI:** 10.1021/acsphyschemau.5c00076

**Published:** 2025-11-11

**Authors:** Patryk Pyrcz, Sylwester Gawinkowski

**Affiliations:** Institute of Physical Chemistry, 119463 Polish Academy of Sciences, Kasprzaka 44/52, Warsaw 01-224, Poland

**Keywords:** single-molecule, SERS, fluctuations, plasmonic nanocavities, cucurbit[7]uril, thionine

## Abstract

Surface-enhanced Raman spectroscopy (SERS) in plasmonic nanocavities enables single-molecule detection through dramatic enhancement of the local electromagnetic field. However, single-molecule SERS (SM-SERS) signals exhibit pronounced fluctuations in both absolute and relative band intensities, as well as abrupt signal dropouts, which complicate reliable analyte detection and identification. A key contributor to this temporal variability is the translational and rotational mobility of molecules within the plasmonic cavity. In this work, we investigated how confining thionine (Th) molecules within the macrocycle cucurbit[7]­uril (CB[7]) suppresses molecular motion and improves spectroscopic stability. We employed two high-field-enhancement geometries — nanoparticle-on-mirror and spherical gold oligomers. The spectral analyses were supported with density functional theory (DFT) calculations and simulations. Our results demonstrate that CB[7] encapsulation improves SM-SERS detection reliability by reducing amplitude fluctuations. Although the average SERS intensity decreases by several tens of percent, signal decay during initial illumination accelerates. Under electronic-resonant excitation of the analyte, detection probability increases owing to the CB[7]-enforced optimal alignment of Th’s transition dipole moment with the nanocavity’s electromagnetic field. Limiting analyte mobility through encapsulation diminishes amplitude fluctuations, while spectral diffusion remains unaffected. These complementary results disentangle two fluctuation mechanisms: molecular motion suppressed by CB[7] and substrate/adatom dynamics unchanged by encapsulation. These findings provide fundamental insights into molecule–nanocavity interactions and establish new strategies for enhancing the reliability of single-molecule detection. The approach opens promising avenues for probing the dynamics of biologically and catalytically relevant species with improved temporal stability and reduced measurement uncertainty.

## Graphical abstract

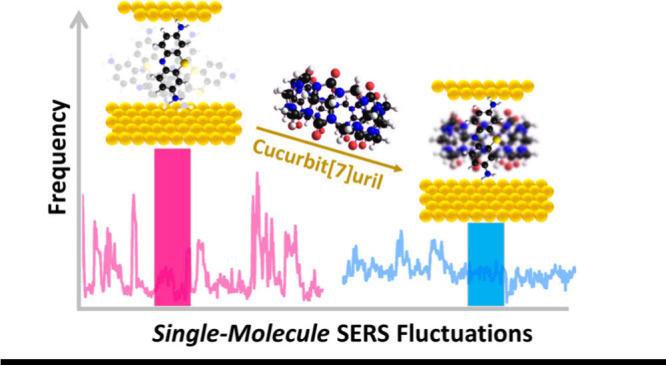

## 1 Introduction

Single-molecule surface-enhanced Raman scattering (SM-SERS) represents one of the most powerful analytical techniques available today, enabling the detection and characterization of individual molecules with exceptional sensitivity and specificity.^1–3^ This extraordinary capability not only elevates established SERS applications — such as disease biomarker detection in biomedical diagnostics,^4,5^ trace contaminant monitoring at the molecular level in environmental analysis,^6^ and direct observation of individual catalytic events^7^ — to their ultimate sensitivity limits, but also unlocks entirely new research avenues that were previously inaccessible with ensemble measurements. The technique can reveal molecular orientation on surface sites,^8,9^ single-molecule conformational dynamics,^10,11^ and transient events that are obscured in ensemble measurements by population averaging, positioning SM-SERS as a revolutionary tool for understanding molecular behavior in unprecedented detail.^3,12^

The exceptional sensitivity of SM-SERS is primarily driven by electromagnetic enhancement through localized surface plasmons — collective electron oscillations in metallic nanostructures induced by light. This plasmonic excitation confines the electromagnetic field within nanometer-scale regions — known as hotspots — thereby boosting the interaction of molecules in these sites with light by orders of magnitude. While this electromagnetic mechanism dominates, the overall SERS process involves additional contributions from chemical enhancement effects, including charge transfer between the analyte and metal surface, changes in electronic resonances, and surface-induced changes in molecular polarizability. When the combined enhancement factors reach about 10 orders of magnitude, single-molecule detection becomes achievable.

A hallmark of SM-SERS measurements is the pronounced temporal fluctuations^13^ of their spectra, which markedly complicates some practical applications. These instabilities manifest as fluctuations in both absolute and relative Raman band intensities, spectral diffusion of peak positions, and intermittent signal dropouts and occasional signal recovery — commonly referred to as “bleaching” and “blinking” events.^14^ Such fluctuations complicate data interpretation, reduce quantitative reliability, and pose substantial challenges for routine analytical applications. The underlying mechanisms are multifaceted, involving translational and rotational diffusion of molecules into and out of hotspots,^8,15^ transient adsorption/desorption events,^16–18^ dynamic reconstructions of plasmonic nanocavities,^9,19^ photochemical reactions,^20,7^ and interactions with surrounding molecules.^21–24^ Understanding both the temporal characteristics and fundamental mechanisms of these processes is crucial not only for developing effective spectral stabilization strategies but also for potentially harnessing these fluctuations as a source of valuable molecular information.

To better understand these complex dynamics, recent studies employing high-speed spectral acquisition have revealed that SM-SERS fluctuations occur over an exceptionally broad temporal range — from seconds down to microseconds^25–27^ — and that substantial differences exist between solution-phase and dry-condition experiments.^24^ In solution-phase experiments, spectral fluctuations are predominantly attributed to adsorption/desorption dynamics of analyte molecules.^24^ In contrast, the mechanisms driving such fluctuations in dry environments remain less clearly defined and warrant further investigation.^24^ This complexity has spurred efforts to stabilize the SERS signal, yet most existing strategies fall short. Common approaches — such as covalent anchoring, self-assembled monolayers, or encapsulation within polymer matrices — often perturb the analyte’s intrinsic Raman signature, introduce unwanted background features, or fail to sufficiently restrict the molecular motions that drive signal fluctuations.^28^ Given the wide-ranging potential applications of SM-SERS, it is essential to improve our understanding of these fluctuation processes and explore new approaches for effectively controlling or at least significantly reducing their occurrence.

Supramolecular encapsulation through host–guest chemistry emerges as a particularly promising yet underexplored strategy that can provide physical confinement without chemical modification of the analyte.^29,30^ Among various supramolecular hosts, cucurbiturils (CB­[n]) stand out due to their unique structural properties: a rigid, pumpkin-shaped architecture featuring an internal hydrophobic cavity capable of encapsulating variety of molecules.^31^ The carbonyl-lined portals of CB­[n] exhibit strong affinity for gold (Au) nanoparticle surfaces, enabling spatial control and robust immobilization of guest molecules within defined plasmonic junctions.^31,32^ The potential of encapsulating analyte molecules in CB­[n] for both SERS and SM-SERS applications has already been demonstrated.^33,34^ However, its impact on SERS spectral stability has not yet been investigated.

To explore this potential, we chose thionine (Th) as a model analyte due to its excellent SERS properties and compatibility with CB[7] encapsulation.^35^ With the Th molecule confined within such a host, one could expect this to be reflected in reduced SM-SERS signal fluctuations. This allows to investigate the effects of molecular confinement on spectral fluctuations, photostability, and single-molecule detection reliability. The presence of carbonyl groups in the CB[7] structure facilitates the adsorption of the macrocycle onto metal surfaces, enabling it to bridge between two Au surfaces while encapsulating the guest molecule, thereby creating well-defined plasmonic junctions (Figure 1). This configuration serves as a model of a SM-SERS hotspot for investigation of signal fluctuations. Our study addresses three key aspects that have not been thoroughly explored previously: the impact of analyte molecule encapsulation by CB[7] on the SERS spectrum in dry and aqueous environment, the photostability of host–guest complex under plasmonic excitation conditions, and the detection and temporal evolution of SM-SERS spectra for the host–guest complex and free molecules at room temperature.

**Figure 1:**
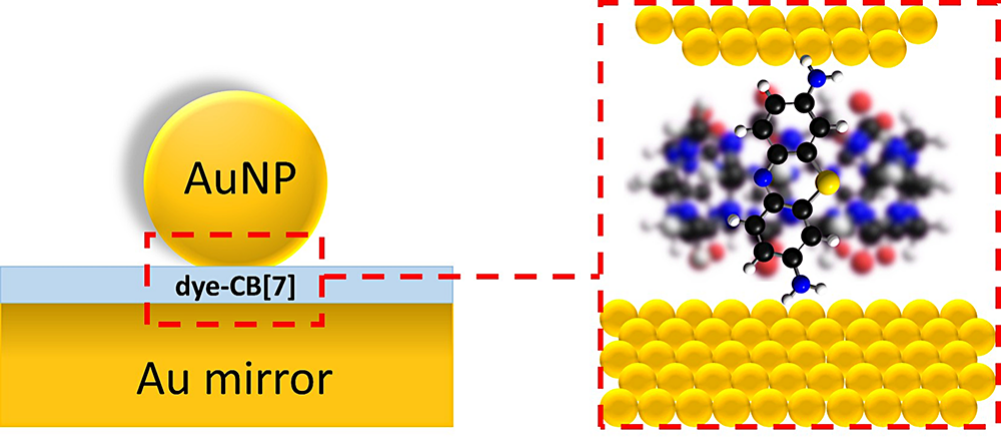
Schematic of the nanoparticle-on-mirror (NPoM) geometry: a spherical Au nanoparticle separated from a Au mirror by a CB[7] monolayer partially loaded with dye molecules.

Our approach integrates experimental characterization with complementary DFT computational simulations to provide fundamental insights into molecule–nanocavity interactions. We compare SERS spectra from free Th molecules and Th–CB[7] complexes using two well-defined, high-field-enhancement geometries: nanoparticle-on-mirror (NPoM) configurations and spherical Au oligomers. These model systems enable systematic investigation of confinement effects on Raman band intensities and temporal spectral characteristics in both aqueous and dry environments.

## 2 Methods

Gold nanoparticles (AuNPs) of ∼50 nm and ∼100 nm diameter were synthesized via a seed-mediated growth protocol based on the citrate reduction method, with sequential addition of HAuCl_4_ and sodium citrate under controlled heating. Plasmonic SERS substrates were prepared using two approaches: (i) aggregation-induced formation of Au oligomers by mixing colloidal AuNPs with thionine (Th) or Th–CB[7] complexes, and (ii) nanoparticle-on-mirror (NPoM) assemblies, in which AuNPs were deposited onto atomically flat Au films functionalized with Th or Th–CB[7]. Flat Au substrates were prepared by epoxy-assisted delamination of thermally evaporated Au from silicon wafers. Raman and SERS spectra were acquired using a Renishaw inVia Raman microscope and previously described custom-built microspectroscopy setup.^36^ Electromagnetic field distributions in oligomer and NPoM nanocavities were simulated using FDTD (Lumerical) with a 0.9 nm interparticle gap and fine meshing in the hotspot region. Quantum-chemical calculations of Th and Th–CB[7] geometries and Raman spectra were performed at the PBE1PBE/Def2TZV level using Gaussian 16. Selected vibrational Raman tensors were analyzed and visualized with custom-written code in Matlab.

Comprehensive descriptions of the experimental methods and simulations are provided in the Supporting Information (SI).

## 3 Results and Discussion

### 3.1 The Impact of Encapsulation on the SERS Spectra

Thionine (Th) was chosen as the guest molecule because it readily forms a stable 1:1 binary complex with CB[7].^35^ Host–guest complex solutions were prepared by mixing aqueous solution of CB[7] and Th (1:1 volume stoichiometry), followed by sonication for approximately 5 min. The encapsulation of Th within the CB[7] molecule was confirmed by absorption spectroscopy (Figure 2). The absorption spectra of Th may display two distinct maxima with concentration-dependent relative intensities attributed to the formation of molecular dimers. The first maximum at 600 nm results from absorption by dissolved monomeric Th, while the second at ∼560 nm, which intensifies with increasing concentration, reflects Th dimer formation at higher concentrations.^37^ Upon encapsulation of Th with an excess of CB[7], the first absorption band undergoes a blue shift from 600 to 593 nm, accompanied by a narrowing of the absorption spectrum. Furthermore, the peak corresponding to dimeric forms becomes significantly less intense. Encapsulation of Th by CB[7] reduces dimer formation — each CB[7] cavity accommodates only one Th molecule — thereby effectively preventing dye aggregation. A control experiment using the larger cucurbit[8]­uril (CB[8]) molecules, which can accommodate two Th molecules, showed an absorption peak at 560 nm with absence of the 600 nm band, confirming that most Th exists in dimeric form (Figure 2).^35,37^

**Figure 2:**
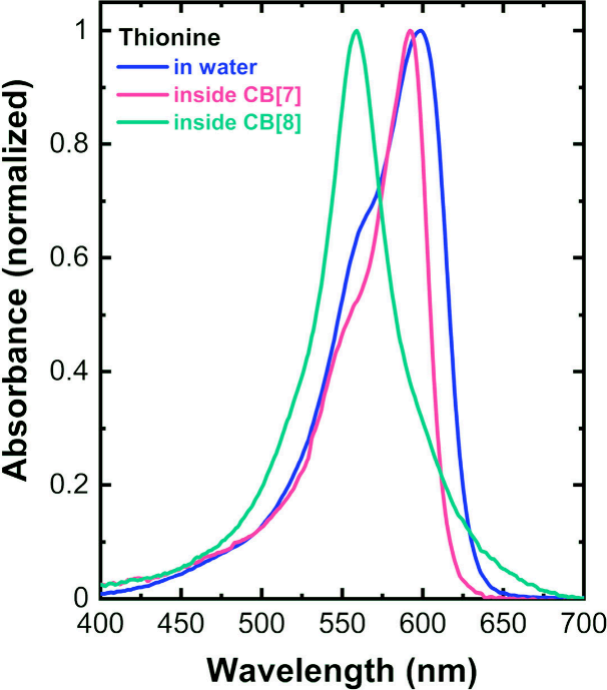
Electronic absorption spectra of 10^–4^ M Th, Th–CB[7], and Th–CB[8] in aqueous solution.

The Th–CB[7] host–guest complex was incorporated into plasmonic cavities formed by spherical Au nanoparticle dimers and larger oligomers. Au nanoparticles have been obtained via seed growth method and characterized by optical spectroscopy and SEM imaging.^38,39^ The Au colloid’s extinction spectrum displayed a surface plasmon resonance peak at 531 nm (Figure S1A, SI) — typical of the dipolar mode of isolated Au nanospheres — while SEM analysis revealed an average sphere diameter of approximately 48 nm (Figure S1B, SI).

Au oligomers were fabricated using the method described by Negru et al.^40^ which involves inducing the aggregation of monomeric Au nanospheres using Th or Th-CB[7], followed by stabilization through the addition of a polymer. The Th–CB[7] complex can attach to Au surfaces via electrostatic interactions at the CB[7] carbonyl portals, effectively “gluing” nanoparticles together.^34,41,42^ For an empty CB[7] cavity, the resulting plasmonic junction height is approximately 0.9 nm, as determined by the dimensions of CB[7].^43^ Nanoparticle aggregation was tracked via extinction spectroscopy: the original single-mode plasmonic peak gradually diminished, while new peaks emerged, reflecting aggregate formation.

In the SERS spectrum of Th, in-plane vibrational modes predominate, although a few out-of-plane modes are also observed (e.g., 427 cm^–1^, band B). A band assignment (Table S1, SI), based on Raman and SERS spectra acquired under different conditions (crystalline and aqueous solution) and excitation wavelengths, together with quantum-chemical calculations, is available in the SI (Figures S4–S6).

SERS measurements of free Th and the Th–CB[7] complex were conducted under nonresonant excitation (785 nm) to isolate the effect of encapsulation on the SERS spectra. The effect of Th encapsulation in CB[7] was studied in anhydrous (Figure 3A) and aqueous environments (Figure 3B). Nonresonant excitation eliminates resonance-induced intensity differences that would arise from CB[7] encapsulation shifting Th’s electronic transition energy. The Th concentration in both samples was maintained at 2 μM, ensuring uniform molecular distribution on the nanoparticle surface. Under preresonant excitation at 633 nm, the SERS spectra of Th and Th–CB[7] show nearly identical relative intensities (Figure S8, SI). The absolute signal levels, however, differ between wet and dry measurements due to variations in optical configuration and laser power.

**Figure 3:**
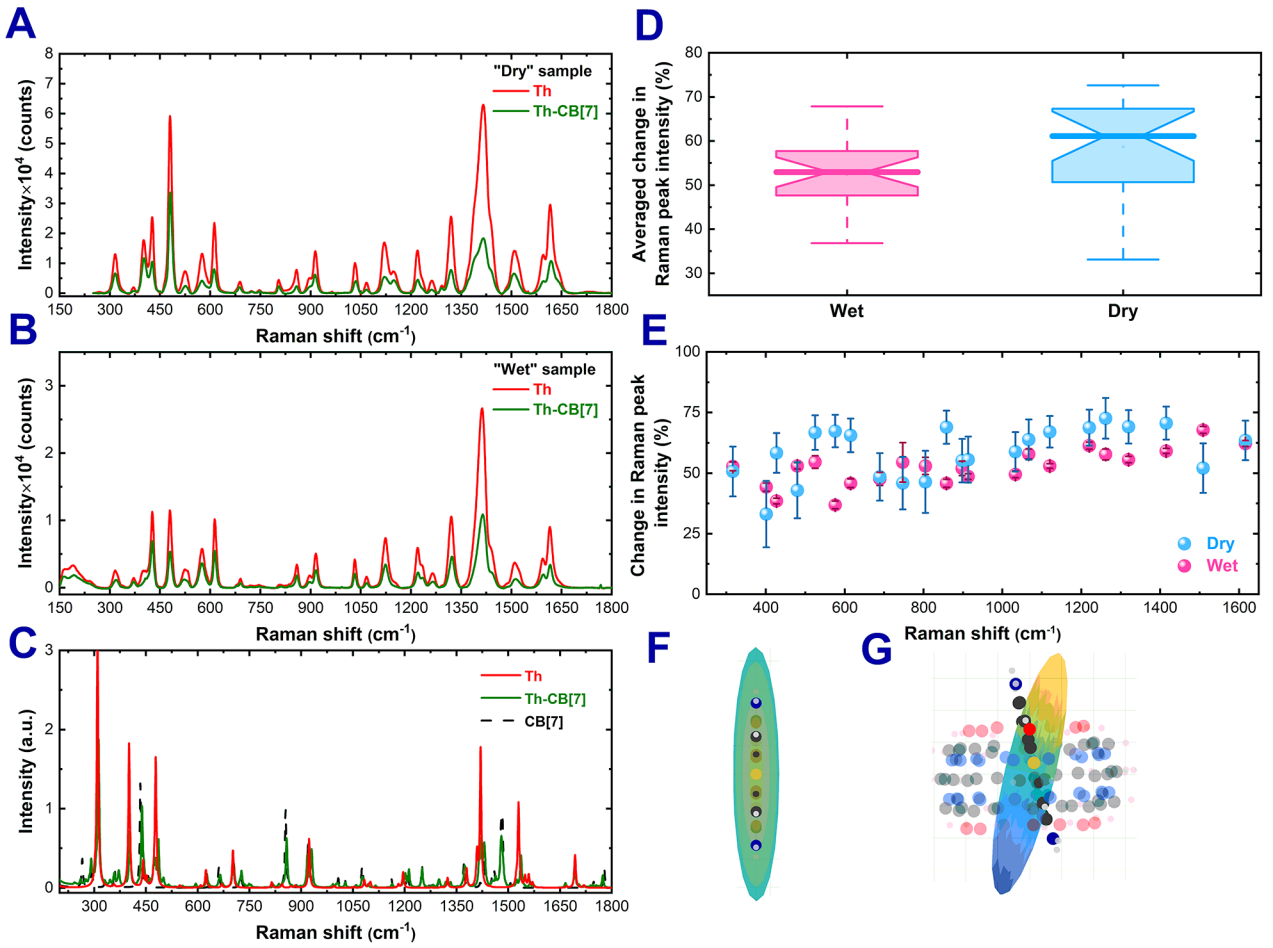
Baseline-corrected average SERS spectra of Th and Th–CB[7] mixed with spherical Au oligomers, measured as dried deposits on glass (A) and in colloidal solution (B). The SERS spectra were recorded using 785 nm excitation. Simulated Raman spectra of Th, CB[7], and Th–CB[7] (C). Averaged percentage change in Th signal intensity (median ± distribution) (D). Change in the intensity of the Th Raman bands for wet and dry environments (E). Visualization of the Raman polarizability tensor for the 316 cm^–1^ mode of free Th (F) and the corresponding tensor for Th encapsulated in CB[7] (G).

Comparative analysis of the bulk SERS spectra for Th and Th–CB[7] reveals that encapsulation does not alter the Raman shift positions of the molecule’s bands — a finding consistent with measurements performed without a plasmonic substrate (Figure S3, SI). However, under off-resonant excitation at 785 nm, in both dry and aqueous environments, most vibrational modes of the encapsulated molecule exhibit lower intensities than free Th, reflecting the attenuating effect of confinement within CB[7]. In contrast, at preresonant 633 nm the reduction is minimal (Figure S8, SI), likely because resonance-Raman enhancement dominates the signal and masks confinement-related modifications of the Raman tensor. This 785 nm intensity reduction trend is slightly milder in aqueous environments (Figure 3D). In dry samples, individual band intensities decreased by approximately 33% at 401 cm^–1^ and up to 73% at 1262 cm^–1^, whereas in wet samples the reduction is from 38% at 427 cm^–1^ to 67.8% at 1509 cm^–1^ (Figure 3E). This comparison indicates that the presence of water partially attenuates the encapsulation-induced loss of SERS enhancement.

The reduction of SERS intensity upon CB[7] encapsulation may arise from several overlapping mechanisms. In principle, the additional spacing introduced by CB[7] could contribute via the strong distance dependence of the near-field enhancement,^44,45^ and steric or cavity-size effects are also known to influence SERS intensities.^46^ However, thionine (∼1.5 nm in length) is longer than the CB[7] cavity (∼0.9 nm), and upon inclusion it adopts an orientation close to perpendicular to the metal surface, as illustrated in Figure 3G. Due to the affinity of the terminal –NH_2_ groups, such an orientation may be preferred irrespective of whether Th is encapsulated by CB[7] or not. In this geometry the effective cavity size is nearly unchanged compared to the free molecule, so a dramatic distance-driven decrease in intensity would not be expected. Indeed, the experimentally observed reduction is moderate (tens of percent), rather than the orders-of-magnitude change that would accompany large variations in cavity size or a perpendicular orientation relative to the plasmonic field. In such a scenario, the SERS spectrum of the Th–CB­[7] complex should display increased intensity of Raman bands whose tensor is associated with in-plane vibrational modes. We found that, apart from the overall decrease in intensity resulting from the inclusion of Th by CB[7], no significant qualitative differences are observed in the SERS spectrum. Moreover, most of the most intense Raman bands in the SERS spectra of both Th and Th–CB[7] correspond to vibrational modes whose Raman tensor is oriented along the long axis of the Th molecule.

DFT simulations and Raman tensor visualizations further support this interpretation. The most Raman-active vibrational modes of thionine have tensors elongated along the molecular long axis, which in the CB[7] complex preferentially aligns with the nanoparticle gap. This alignment preserves efficient coupling to the plasmonic field, whereas a flat-lying geometry would result in negligible enhancement due to tensor–field misalignment. Taken together, these results indicate that the hotspot size is essentially unchanged by encapsulation and that the observed decrease in intensity reflects confinement- and orientation-induced changes in the Raman tensor, with distance and steric effects providing secondary contributions.

The observed Raman peak intensity changes are largely consistent with simulated spectra (Figure 3C). The variation in Raman band intensity resulting from CB[7] encapsulation may be associated with changes in the Raman tensor caused by alterations in molecular structure due to spatial constraints within the CB[7] cavity and interactions between the Th molecule and CB[7]. Such changes are visualized for the 316 cm^–1^ mode Raman tensor in Figure 3F and G. DFT simulations show that the Th molecule inside CB[7] is slightly tilted relative to the longest axis of the CB[7] skeleton, consistent with previous reports,35 although the change in tensor tilt direction is opposite. The effect of dye encapsulation in CB[7] on SERS spectral intensities has been demonstrated for rhodamine 6G (R6G), with Taylor et al.^34^ reporting that encapsulation causes disappearance of lower-wavenumber vibrational modes due to vibrational restriction within the CB[7] cavity.

### 3.2 The Photostability of the Host–Guest Complex in a Plasmonic Environment

The photostability of Th and Th–CB[7] was compared in two different plasmonic configurations — nanoparticle-on-mirror (NPoM) and spherical Au oligomers — both of which offer strong electromagnetic field confinement allowing SM-SERS detection.^5,8^ To prepare NPoM samples, smooth Au mirrors were immersed in aqueous Th or Th–CB[7] solutions for at least 24 h and subsequently coated with spherical Au nanoparticles. As a result, plasmonic nanocavities formed between the mirror surface and nanoparticle, trapping the analyte molecules within the gap. Spherical Au oligomers were generated by aggregating nanoparticles in the presence of Th or Th–CB[7]. The resulting colloids were then deposited onto glass substrates and air-dried. Detailed procedures are provided in the SI. Two diameters of spherical Au nanoparticles — 48 and 97 nm (Figures S1C and S1E, SI) — were employed to tune the plasmonic cavities spectrally close to 633 and 785 nm excitations, respectively. The 633 nm excitation overlaps the dye’s electronic transition, whereas the 785 nm excitation is off-resonance.

For each sample series, SERS raster maps were acquired through consecutive measurement cycles. Each SERS map was averaged to a single spectrum and, after baseline correction, the peak height at 483 cm^–1^ was extracted as the photodegradation metric. This spatially averaged SERS signal intensity showed a systematic decrease across successive measurements. Figure 4 displays the temporal evolution of normalized SERS intensities with corresponding exponential decay fits. This intensity reduction likely stems from photodecomposition of analyte molecules within hotspots or their diffusion away from these regions due to strong electromagnetic fields. The decay kinetics reveal distinct behaviors between sample types. NPoM samples exhibit monoexponential decay characteristics, while Au oligomers require biexponential fitting to accurately describe the experimental data. This difference in decay complexity reflects the underlying structural variations: oligomers possess diverse morphologies generating hotspots with varying electromagnetic field strengths, whereas NPoM structures demonstrate greater uniformity. The similar energy densities across NPoM hotspots result in homogeneous photodegradation kinetics, manifesting as single exponential decay. In contrast, the heterogeneous hotspot distribution in oligomer samples leads to multiple decay components, reflecting the range of field enhancement factors and corresponding photodecomposition rates.

**Figure 4:**
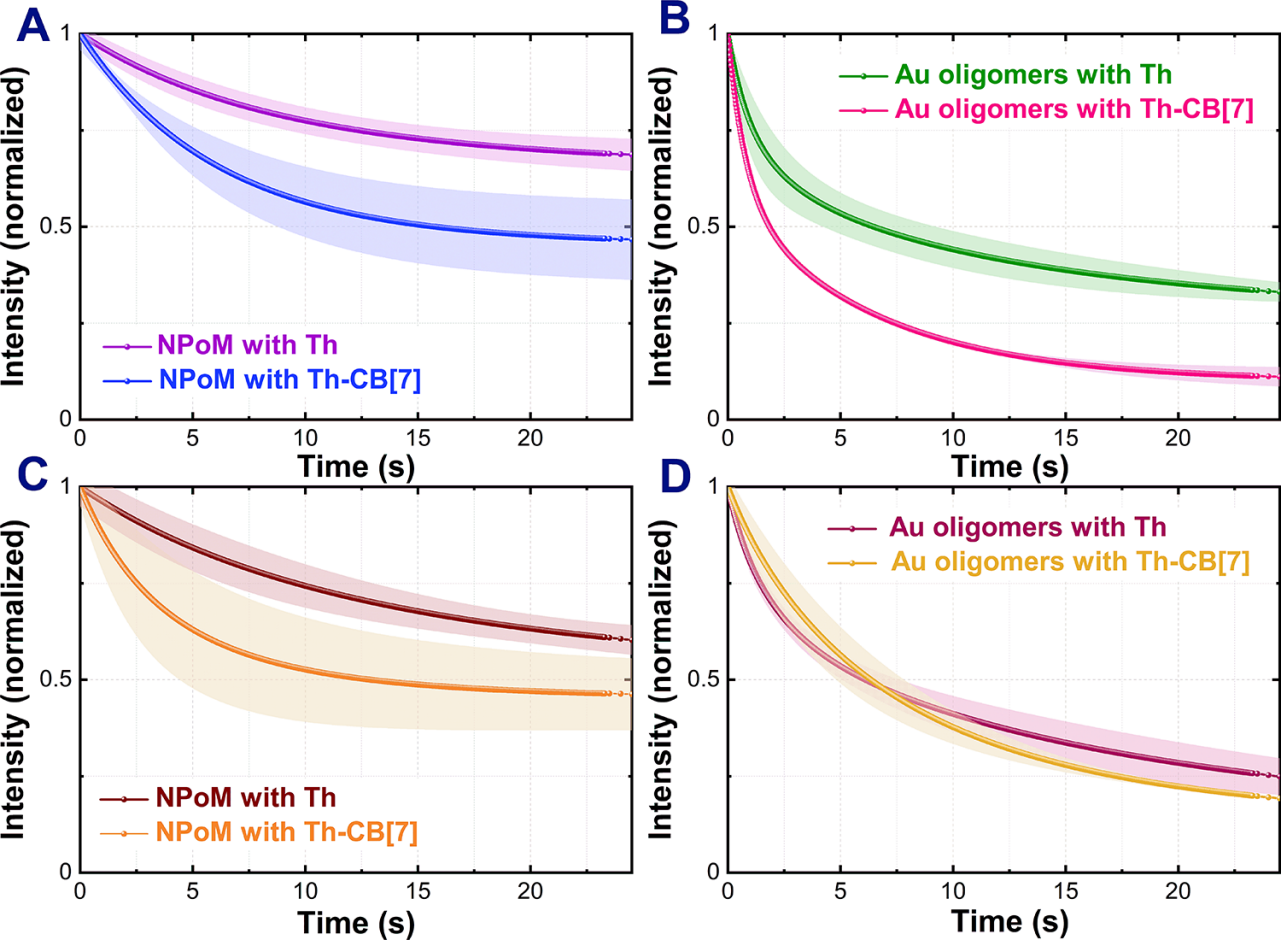
SERS signal decay profiles for Th and Th-CB[7] registered from NPoM excited at 633 nm (A) and at 785 nm (C), and from Au oligomers excited at 633 nm (B) and 785 nm (D). Each curve represents the baseline-corrected signal averaged over a 20 × 20 μm SERS map (1 μm step) and fitted with a monoexponential decay (A, C) or a biexponential decay (B, D). Measurements were performed for different areas of samples with 0.5 s integration time per spectrum and laser powers of 17 μW at 633 nm or 60 μW at 785 nm. Standard deviations are indicated as shaded regions around each curve; technical replicates across distinct regions (*n* = 5 NPoM, *n* = 3 Au oligomers), 50 maps per region. Corresponding decay fits are listed in Table S2 (SI).

In both the NPoM and Au oligomer configurations, Th–CB[7] displayed a more rapid SERS signal decay than Th, regardless of the excitation wavelength. This apparently paradoxical acceleration of photodegradation can be rationalized by more efficient and homogeneous electromagnetic coupling of the stabilized Th–CB[7] complex with the plasmonic hotspot. The enforced orientation inside CB[7] enhances the overlap between the molecular transition dipole and the cavity field, increasing absorption and accelerating photochemical decay. Thus, supramolecular stabilization inherently involves a trade-off: improved signal stability at the cost of reduced photostability.

To quantify the degradation rate, we compared the SERS decay profiles after 11.5 and 23.5 s of continuous illumination. After 11.5 s of illumination at 633 nm, the signal of the NPoM sample decreased by 25% for Th and 46% for Th–CB[7]. Under 785 nm excitation, the signal reductions were 28% for Th and 50% for Th–CB[7], respectively. After 23.5 s of illumination, the signal losses under both resonant and nonresonant conditions were more pronounced. However, the rate of decay was slower compared to the initial 11.5 s period. In the case of Au oligomer cavities, the largest decrease in SERS signal under resonant conditions was observed after 11.5 s of illumination, amounting to 58% for Th and 82% for Th–CB[7]. Under nonresonant conditions at the same time point, the signal decay values for both systems were similar and within the margin of error. After 23.5 s of illumination, the SERS signal decay further increased. Nonetheless, as in the NPoM cavity, the rate of SERS signal degradation was less dynamic than during the first 11.5 s of exposure.

The kinetic variability of SERS signal decay in plasmonic nanocavities is evident in both qualitative and quantitative analyses. Table S2 (SI) summarizes the kinetic parameters obtained from fitting the experimental decay curves. For NPoM samples, a monoexponential model generally sufficed, whereas for oligomeric cavities a biexponential model typically provided a better fit. These results highlight that the NPoM structures provide more homogeneous decay kinetics, whereas the oligomeric assemblies reflect greater heterogeneity of hotspot populations. Taken together with the trends discussed above, the quantitative analysis reinforces the conclusion that CB[7] encapsulation accelerates SERS signal decay, while cavity morphology governs the kinetic complexity.

The significantly faster SERS signal decay observed upon inclusion of the molecule within CB[7] can be attributed to constrained molecular orientation that improves the coupling of Th’s transition dipole with the cavity field, thereby increasing effective field exposure and accelerating photochemical decay. The CB[7] macrocycle, featuring carbonyl groups at both portals, forms stable electrostatic interactions that preferentially position the host–guest complex within nanocavities between adjacent nanostructures. This selective positioning ensures consistent placement in regions of maximum electromagnetic field enhancement, resulting in strong and reproducible SERS signals. Moreover, host encapsulation constrains the guest’s orientation so that Th’s long axis aligns with the electromagnetic field. Because the molecule’s transition dipole moment also lies along this axis, the CB[7]-encapsulated Th undergoes more efficient electronic excitation. In contrast, free Th molecules exhibit considerably more diverse binding configurations with nanostructures, with positioning not necessarily optimized for the nanocavity center. Furthermore, unlike the CB[7]-encapsulated complex, free Th molecules can adopt various orientations relative to the electromagnetic field direction within the cavity, leading to greater heterogeneity.^47^ The constrained geometry imposed by CB[7] encapsulation thus promotes more uniform and intense SERS responses, albeit at the cost of increased photodegradation rates due to consistently higher field exposures.

The experimental findings are further corroborated by finite-difference time-domain (FDTD) simulations (Figures S9 and S10, SI). These calculations show that electromagnetic enhancement factors in dimer cavities are higher than in NPoM configurations, which aligns with the faster signal decay rates observed in oligomeric systems. Moreover, actual enhancement factors in real samples may exceed these values, since nonideal nanoparticle shapes — surface faceting and other deviations from perfect sphericity — can generate even stronger localized fields at sharp edges and vertices.^48^

### 3.3 The Influence of Host–Guest Complexation on SM-SERS Spectra

The SM-SERS of Th and the Th–CB[7] complex was carried out in a NPoM configuration at room temperature under molecular preresonance excitation (λ = 633 nm). We found that encapsulation of Th by CB[7] markedly enhances its detectability (Figure 5), both by increasing the overall number of SERS-active hotspots and by raising the fraction of those that can be classified as single-molecule events. In NPoM cavity samples, SERS mapping showed about a 4-fold increase in the frequency of SERS-active hotspots for the Th–CB[7] complex compared to free Th. Classification of SM-SERS events was based on the temporal evolution of the signal, with the sudden disappearance of a trajectory taken as the criterion. Among all SERS-active hotspots, ∼ 75% could be classified as single-molecule events in the case of Th–CB[7], whereas for free Th the proportion was ∼ 55%.

**Figure 5:**
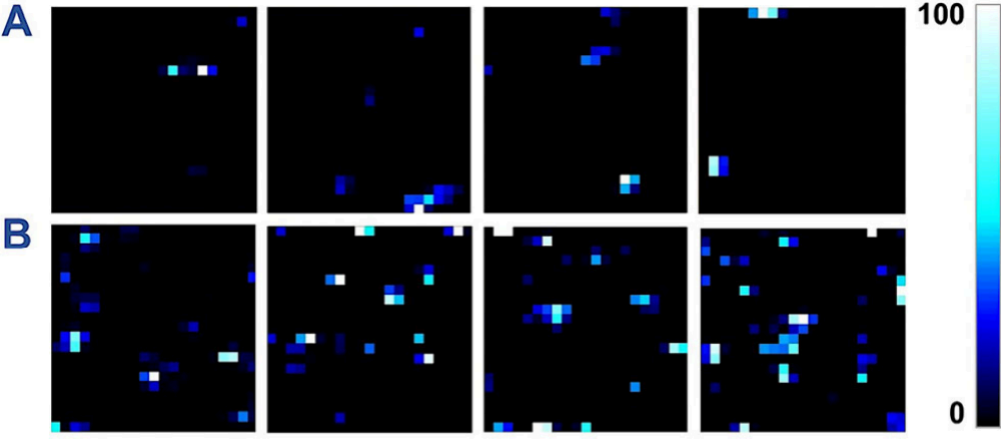
SERS maps (10 × 10 μm) of an NPoM for Th (A) and Th–CB[7] (B), recorded with 633 nm excitation at 17 μW laser power, 0.5 s integration time per pixel, and a 0.5 μm step size.

This higher detectability likely arises from the guest–host complex’s orientation within the NPoM hotspot. Encapsulation of Th by CB[7] enforces alignment of its transition dipole moment with the nanocavity’s electric-field vector, thereby enhancing the resonance Raman intensity to levels sufficient for single-molecule detection. In contrast, free Th molecules can adopt orientations in which their transition dipole moments are perpendicular to the cavity field, preventing them from benefiting fully from preresonance excitation and yielding much weaker not detectable signals. DFT calculations indicate that, even under nonresonant excitation, the principal axes of the Raman polarizability tensors for Th’s most intense Raman-active modes form ellipsoids whose longest axes coincide with the molecule’s long axis. Because this tensor orientation is misaligned with the nanocavity’s electric-field vector, Raman scattering is inefficient. The Th molecule has two amino groups at its termini that can bind to the two Au surfaces lining the cavity, promoting its vertical alignment. However, these two amino groups can also interact with the same Au surface, an interaction further enhanced by the sulfur atom’s affinity for Au.^49^ The comparison with the Th–CB[7] complex shows that, although this orientation of free Th is not strongly favored, it still occurs with measurable frequency in the SM-SERS maps.

The SERS measurements within NPoM revealed distinct temporal behaviors for encapsulated and free molecules. Figure 6 presents spectral evolution under preresonance excitation (633 nm) for single Th-CB[7] complexes (Figure 6A) and free Th molecules (Figure 6B). Additional trajectories are shown in Figure S11 (SI).

**Figure 6:**
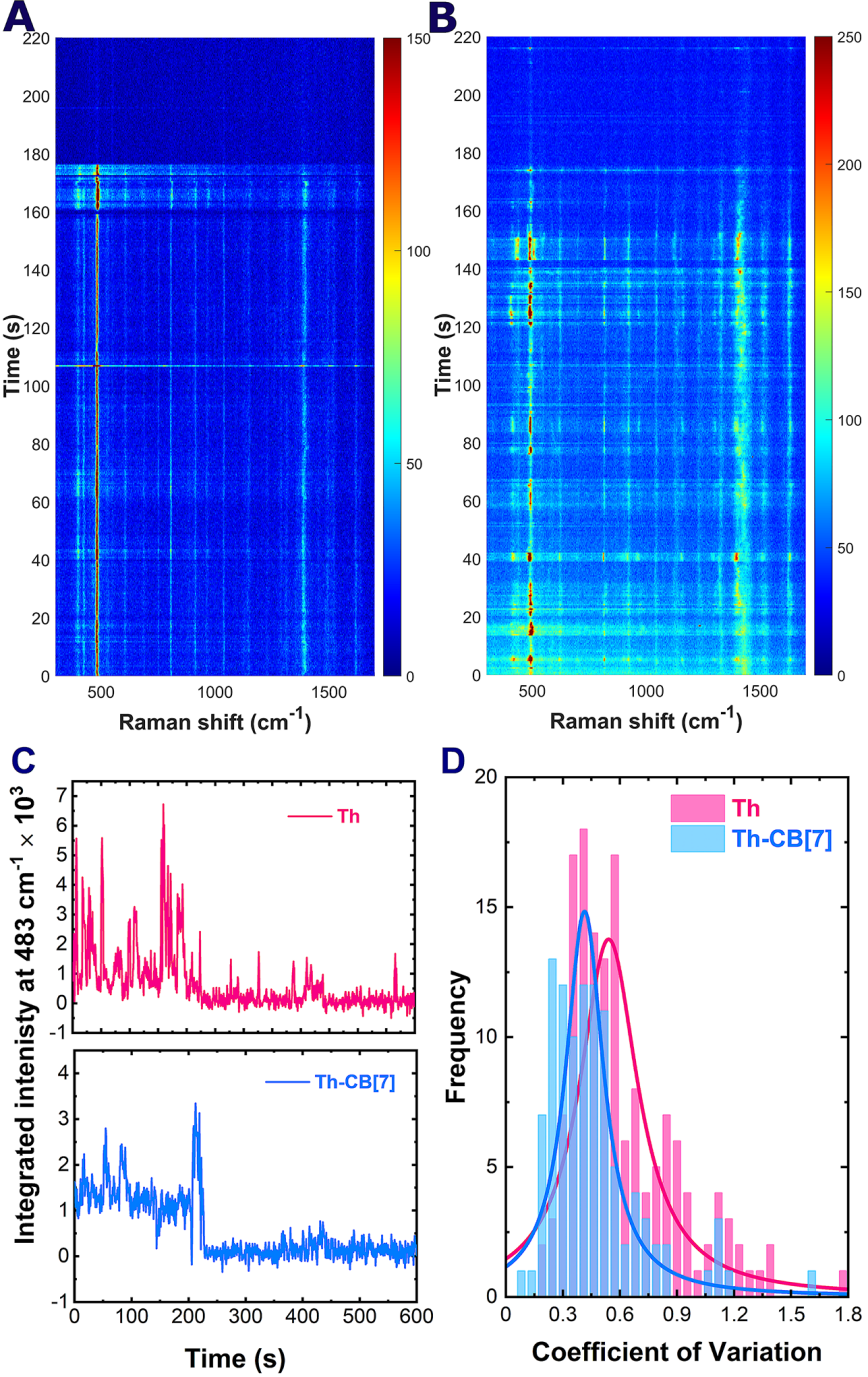
Temporal evolution of SERS spectra for a single Th–CB[7] complex (A) and a single free Th molecule (B), recorded with 0.5 s integration and 633 nm excitation. Extracted peak-area traces of the 483 cm^–1^ band for Th–CB[7] and free Th from panels A and B (C). Histograms of the coefficient of variation for free Th and Th-CB[7] samples, each fitted with a Lorentzian function (D).

Both systems exhibit characteristic intensity fluctuations inherent to the single-molecule regime. However, free Th molecules displayed exceptionally pronounced intensity bursts lasting less than one second. Given high molecular mobility under intense electromagnetic fields and limited temporal resolution (CCD-constrained), we attribute these signals to averaged responses from rapidly interconverting orientations within the hotspot. In contrast, Th-CB[7] complexes demonstrated significantly more stable profiles with reduced fluctuation amplitudes. Both systems ultimately underwent photobleaching — sudden, irreversible signal loss (Figure 6C). These observations are typical for SM-SERS spectra and have been extensively reported in the literature.^8,13,46,50,51^

The pronounced differences in the temporal behavior of freely adsorbed Th and Th complexed with CB[7] indicate distinct mobilities of the molecules within the hotspot. Free Th molecules near the hotspot exhibit higher mobility, as their rotational and translational motions are largely unrestricted. In contrast, encapsulation within CB[7] markedly reduces molecular mobility: the host acts as a molecular cage that constrains reorientation and suppresses translational freedom through electrostatic anchoring to the Au surface. Consequently, Th–CB[7] produces a stable temporal profile in the SM-SERS signal, whereas free Th molecules display a more dynamic temporal evolution.

The diversity of the dynamics of the temporal profiles of the SM-SERS signal is mirrored in the analysis of the coefficient of variation (CoV), which represents a ratio between the standard deviation of the intensity signal and its mean value. Analysis of the CoV revealed the increased intensity fluctuations in SM-SERS spectra evolution observed for freely adsorbed Th molecules compared to the Th-CB[7]. The analysis of over 100 individual single-molecule events for each system, fitted with Lorentzian distributions (Figure 6D), revealed a mean CoV of 0.54 for free Th versus 0.41 for Th–CB[7], corresponding to a 24% reduction in relative intensity variability upon encapsulation. Furthermore, the CoV distribution for Th exhibits a noticeably broader spread, indicating greater heterogeneity in fluctuation behavior across the molecular population.

The elevated CoV for free Th directly correlates with enhanced molecular mobility, stemming from unrestricted rotational and translational degrees of freedom within the hotspot. The molecule can sample diverse orientations relative to the local field polarization and explore different spatial positions within the hotspot volume, each configuration contributing distinct enhancement factors to the time-averaged signal. Conversely, Th encapsulation within CB[7] imposes severe geometric constraints on molecular motion through the restrictive nanoscale cavity environment. The encapsulated molecule cannot undergo reorientation from perpendicular to parallel alignments relative to the nanocavity axis, assuming the CB[7] host maintains fixed orientation on the surface. The electrostatic interactions between CB[7]’s carbonyl portals and the Au surface create stable anchoring points that prevents translational motion of the host–guest complex, confining the system to a fixed spatial location within the hotspot.

These results align with previous investigations by Lindquist et al.,^24^ who demonstrated molecule-dependent CoV variations, reporting mean values of 0.56 for covalently bound molecules versus 0.65 for physisorbed species both registered in a dry environment. Our results support the conclusion that SM-SERS spectral fluctuations depend fundamentally on molecular binding characteristics and surface affinity, with stronger surface interactions generally correlating with reduced temporal variability.

In addition to the intensity fluctuations, we observe spectral diffusion in vibrational Raman modes, providing further compelling evidence for single-molecule detection. Our measurements reveal sudden, discrete frequency shifts in Raman bands that persist for extended periods before abruptly returning to their original positions or shifting to new frequencies (Figure S11, SI). We found that spectral fluctuations of Raman band positions are not independent but are accompanied by concurrent intensity variations. These observations point to a complex mechanism underlying the fluctuations in SM-SERS spectra. Unlike freely adsorbed molecules on metal surfaces, CB[7] encapsulation markedly restricts both translational and rotational motion. Although Th effectively occupies the CB[7] cavity, its packing coefficient of less than unity indicates residual free volume, allowing limited conformational flexibility and small-amplitude motions within the host cavity.

To elucidate the underlying fluctuation mechanisms, we examined the relationship between peak-position and peak-intensity variations. We performed Spearman correlation analysis on four pairs of strongest Th Raman bands. The population comprised over 100 SERS spectra recorded from single molecules. Prior to analysis, experimental data underwent preprocessing including noise reduction, spectral smoothing using the Savitzky–Golay filter, and Lorentzian fitting of selected Raman bands. The analysis reveals fundamentally different correlation behaviors between these two parameters (Figure 7). Peak amplitude variations exhibit strong correlations (mean values: 0.67–0.87) with narrow, reproducible distributions, suggesting a unified enhancement mechanism governing intensity fluctuations across different bands. In contrast, peak position correlations are weaker (0.19–0.67) with notably broader distributions, indicating that spectral diffusion involves more dynamic or heterogeneous processes than amplitude fluctuations.

**Figure 7:**
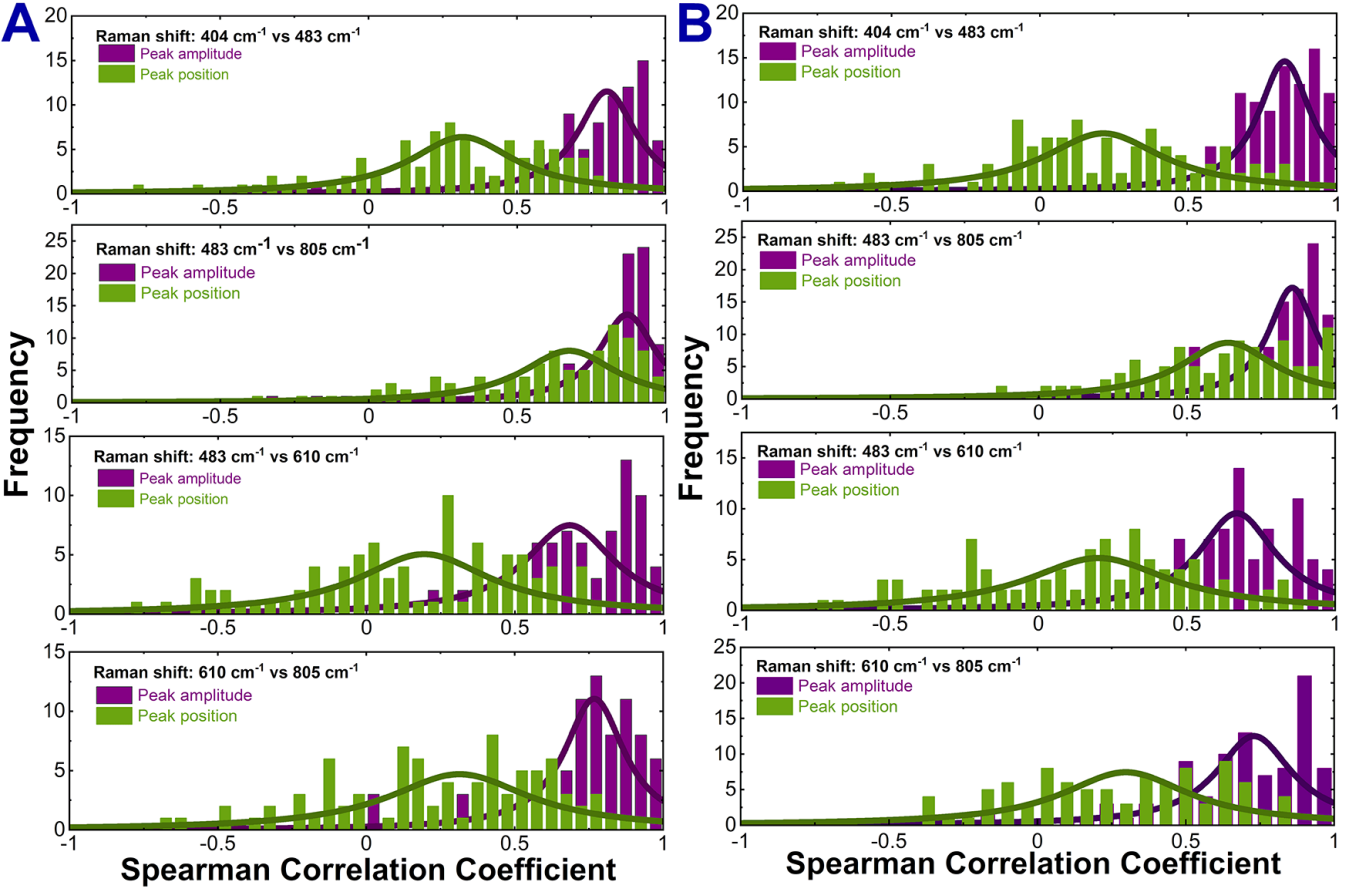
Distributions of Spearman’s correlation coefficients between Raman peak-position fluctuations and SERS-signal amplitude changes for single-molecule SERS spectra of free Th (A) and Th–CB[7] (B) for selected pairs of Raman bands. Each distribution was fitted with a Lorentzian function. Sample sizes corresponding to each pair of Raman modes — 404 cm^–1^ vs 483 cm^–1^, 483 cm^–1^ vs 610 cm^–1^, 483 cm^–1^ vs 805 cm^–1^, and 610 cm^–1^ vs 805 cm^–1^ — were as follows: *n* = [87, 87, 99, and 87] for Th, and *n* = [100, 95, 107, and 92] for Th–CB[7].

This distinct correlation behavior suggests that SM-SERS fluctuations arise from at least two largely decoupled mechanisms. The strong amplitude correlations likely reflect coherent electromagnetic enhancement variations affecting all bands simultaneously, while the weak position correlations indicate that spectral diffusion involves mode-specific chemical interactions. Notably, Th encapsulation within CB[7] leaves these correlation patterns largely unchanged: mean correlation coefficients are similar for free and encapsulated molecules, indicating that the underlying fluctuation mechanisms persist.

The correlation analysis indicates that CB[7] encapsulation does not eliminate spectral- or intensity-type fluctuations. Importantly, the unchanged correlation patterns upon CB[7] encapsulation do not contradict the CoV analysis (Figure 6). Rather, the two results highlight distinct fluctuation mechanisms. The reduction in CoV reflects the suppression of slow, large-scale translational and rotational motion by CB[7], whereas the preserved correlation patterns indicate that fast, local adatom dynamics remain unaffected. Taken together, these results establish that SM-SERS instabilities originate from two largely decoupled mechanisms — molecule-driven fluctuations, which are suppressed by encapsulation, and substrate-driven fluctuations, which persist regardless of confinement. We observe a notable reduction in the CoV for intensity fluctuations in the encapsulated system (Figure 6D), suggesting restricted molecular mobility within the CB[7] cavity. This outcome suggests that encapsulation primarily affects the frequency of amplitude variations, as the Raman peak amplitude served as the probe parameter in the CoV analysis. This observation aligns with crystallographic studies showing that CB­[n] hosts undergo ellipsoidal deformations upon guest inclusion.^52^ Molecular dynamics simulations by El-Barghouthi et al.^53^ of methyl viologen in CB[7] revealed that while guest rotation within the cavity remains possible, it induces structural deformations in the CB[7] diameter. Our experimental results support these computational predictions, though direct observation of such dynamics would require substantially shorter acquisition times combined with increased excitation power to maintain adequate signal-to-noise ratios.

Spectral jumps are a hallmark of SM-SERS, as ensemble measurements average out such individual molecular events. The underlying mechanisms of spectral diffusion remain unclear, with several theories proposed in the literature. Benz et al.^19^ introduced the concept of picocavity formation, in which transient interactions between mobile Au adatoms on the nanoparticle surface and analyte molecules create highly localized electromagnetic hotspots that dynamically enhance specific vibrational modes. Park et al.^54^ applied statistical correlation analysis to fluctuating vibrational modes of malachite green and demonstrated that spectral diffusion may originate from molecular rotational dynamics and fluctuations in the local electromagnetic environment. Recently, Ai et al.^55^ used SERS to probe dynamic changes of CB[7] and encapsulated molecules. Their experiments involved CB[7]-based plasmonic molecular junctions formed in solution by adsorption of Au nanoparticles onto CB[7]-modified Au nanoelectrodes.^55^ SM-SERS trajectories of aminoferrocene complexed with CB[7] revealed sudden Raman shift transitions between 1080 and 1105 cm^–1^, which were attributed to rotational motions of the aminoferrocene molecule within the CB[7] cavity. Together, these studies support the explanation for our observations: CB[7] selectively suppresses translational/large-angle reorientational motion (captured by the reduced CoV), while residual rotational degrees of freedom and adatom dynamics continue to drive spectral diffusion (consistent with the unchanged correlation patterns).

The dynamic nature of Raman peak position shifts provides further insight. We propose that these spectral fluctuations originate from transient interactions between Au adatoms within the plasmonic cavity and the analyte molecule, in line with the picocavity model of Benz et al.^19^ The transient formation and breakup of adatom–molecule complexes induce Raman band frequency shifts, ranging from subtle to pronounced, as a result of changes in the local chemical environment. This mechanism accounts for the broad distribution of position correlation coefficients observed in our data, as adatom dynamics are inherently stochastic and highly sensitive to local environmental conditions. Our experimental results are consistent with these molecular dynamics predictions. However, direct observation of rotational dynamics would require substantially shorter spectral acquisition times combined with higher excitation powers to maintain an adequate signal-to-noise ratio. In addition, temperature-dependent studies could provide further insight into the roles of adatom dynamics and molecular motion within the hotspot in SM-SERS spectral fluctuations. Such experiments would help disentangle the relative contributions of adatom migration and molecular mobility, thereby yielding a clearer mechanistic picture of spectral diffusion in plasmonic nanocavities.

## 4 Summary and Conclusions

This study presents a comprehensive investigation of single-molecule surface-enhanced Raman scattering (SM-SERS) with a focus on influencing molecular dynamics through supramolecular encapsulation. Using thionine (Th) as a model system, we demonstrated that encapsulation within cucurbit[7]­uril (CB[7]) provides a simple approach to increase stability of SM-SERS signals while preserving the intrinsic characteristics of single-molecule detection and suppressing molecular dimerization. Encapsulation within CB[7] resulted in a marked bulk SERS intensity reduction without shifting Raman band positions, reflecting conformational constraints and altered polarizability tensors as supported by DFT simulations. Time-resolved single-molecule SERS (SM-SERS) measurements revealed distinctly different fluctuation patterns between free and encapsulated molecules. Free Th exhibited rapid intensity ‘jumps’ with high variability, whereas Th–CB[7] displayed more stable temporal behavior with a lower coefficient of variation across single-molecule trajectories. Analysis of single-molecule temporal evolution revealed strong correlations between amplitude fluctuations but weaker position correlations, suggesting distinct underlying mechanisms governed by the picocavity model involving transient Au-adatom interactions.

The supramolecular encapsulation within CB[7] provides effective physical confinement without chemical modification of the target molecule, offering a promising alternative to traditional covalent anchoring approaches that often alter molecular properties. The mechanism involves CB[7] acting as a molecular ″cage″ that restricts both translational and rotational motion of the guest molecule, leading to several interconnected effects that collectively improve SM-SERS performance. The enforced molecular orientation within the CB[7] cavity has multiple consequences for single-molecule detection. It aligns the molecular dipole moment more favorably with the electric field in nanocavities, resulting in several-fold increased detection frequency and making SM-SERS measurements more reliable and accessible. Simultaneously, this stabilized orientation reduces unwanted signal fluctuations while preserving characteristic SM-SERS phenomena including blinking and bleaching, confirming that the fundamental single-molecule nature is maintained. The confined geometry also leads to more predictable and reproducible SERS signals. However, the study reveals a paradoxical trade-off inherent to this stabilization approach. While encapsulation reduces signal fluctuations, it accelerates photodegradation due to more efficient and homogeneous electromagnetic coupling between the stabilized molecule and the plasmonic hotspot. This observation highlights the complex interplay between molecular confinement, electromagnetic enhancement, and photochemical processes, emphasizing the need for optimized measurement protocols that balance signal stability with photostability.

Limiting analyte mobility through encapsulation diminishes amplitude fluctuations, while correlation analysis shows that spectral diffusion remains unaffected. These complementary results disentangle two possible fluctuation mechanisms: molecular motion suppressed by CB[7] and substrate/adatom dynamics unchanged by encapsulation.

The results establish a foundation for advancing both fundamental understanding and practical applications of stabilized SM-SERS. Future work should focus on investigating different supramolecular hosts to optimize the stabilization-enhancement balance, developing faster acquisition protocols to minimize photodegradation, and implementing this approach in quantitative analytical applications where signal reproducibility is critical. The controlled manipulation of single-molecule dynamics through supramolecular chemistry represents a step forward in making SM-SERS a more robust and predictable analytical technique, ultimately bridging the gap between fundamental research and practical implementation in fields requiring sensitive and reproducible molecular detection.

## Supplementary Material



## Data Availability

Data for this article are available at RepOD repository at 10.18150/Z6XLJ1.

## Notes

Experimental details, materials and methods, including sample fabrication procedures, experimental setup, theoretical calculations; additional results, including nanoparticles characterization, Raman and SERS spectra, fitting parameters, FDTD simulations, examples time traces of SM-SERS signals (PDF)

## Abbreviations

SERS: Surface-enhanced Raman spectroscopy
SM-SERS: single-molecule SERS
Th: thionine
CB­[7]: cucurbit­[7]­uril
CB­[8]: cucurbit­[8]­uril
DFT: density functional theory
AuNP: gold nanoparticle
NPoM: nanoparticle-on-mirror
FDTD: finite-difference time-domain
