# Supplementary material for: Supramolecular Stabilization of Single-Molecule SERS: Cucurbit[7]uril Encapsulation of Thionine
Source: ACS Phys Chem Au. 2025 Nov 11;6(1):57–68. doi: 10.1021/acsphyschemau.5c00076 (PMC12856645; doi:10.1021/acsphyschemau.5c00076)
Supplement: Supplementary file 1 [file pg5c00076_si_001.pdf]

Supplementary Information

Supramolecular Stabilization of Single-Molecule SERS: Cucurbit[7]uril Encapsulation of Thionine

Patryk Pyrcz* and Sylwester Gawinkowski*

Institute of Physical Chemistry, Polish Academy of Sciences,

01-224 Warsaw, Kasprzaka 44/52, Poland

**E-mail: ppyrcz@ichf.edu.pl, sgawinkowski@ichf.edu.pl*

Table of contents

I. Experimental

1. **Materials**
2. **Synthesis protocols**
3. **Characterization methods**
4. **Preparation of plasmonic nanocavities**
    a. Au oligomers with Th or complexes Th-CB[7]
    b. Nanoparticle on mirror (NPoM) with Th or complexes Th-CB[7]
5. **Raman and SERS measurement**
6. **Numerical simulations**
7. **Quantum-chemical calculations**

II. Additional results

1. **Characterization of Au nanoparticles**
2. **Encapsulation of thionine in cucurbit[7]uril**
3. **Raman and SERS characteristics of thionine with varying excitation wavelengths**
    a. Raman and SERS spectra of Th
    b. Raman and SERS bands assignments
4. **Pre-resonance (excitation wavelength 633 nm) SERS of Th and Th-CB[7] in a dry and wet environments**
5. **SERS signal decay profiles for Th and Th-CB[7] – kinetic parameters**
6. **Numerical simulations**
7. **Temporal evolution of SM-SERS spectra for Th and Th-CB[7] recorded in the NPoM cavity**

III. References

I. Experimental

1. Materials

Gold(III) chloride trihydrate (≥99.9% trace metals basis), sodium citrate dihydrate (99%), thionine acetate (Th) (≥85 %), cucurbit[8]uril hydrate (>99.5 %), and polyvinylpyrrolidone (average M_w ~55,000) were purchased from Sigma-Aldrich. Cucurbit[7]uril hydrate (>99.5%) was purchased from Strem Chemicals, Inc. Acetone (HPLC, 99.9%), hydrogen peroxide (30%), nitric acid (63%), hydrochloric acid (38%) and sulphuric acid (98%) were purchased from CHEMPUR. All reagents were used as purchased. Water used in the experiments was deionized (Elix, Millipore, >15 MΩ·cm^{-1}). All glassware were thoroughly cleaned using aqua regia before nanoparticle synthesis and then rinsed with deionized water.

2. Synthesis protocols

Spherical gold nanoparticles (AuNPs) were prepared through a seed-mediated growth approach following literature procedures.[1,2] The initial seed particles were generated using the citrate reduction technique originally developed by Turkevich *et al.*[3]

The first synthesis aimed to obtain AuNPs with a diameter of approximately 50 nm. The first step included seed preparation. In this process, sodium citrate solution (340 mM, 0.5 mL) was introduced into a vigorously stirred, boiling $HAuCl_4$ solution (0.254 mM, 99.5 mL) under reflux condenser. The resulting mixture was maintained at 90°C for 30 minutes. To facilitate seed particle growth, stock solutions containing $HAuCl_4$ (25 mM) and sodium citrate (60 mM) were freshly prepared. During the initial growth phase, the seed solution (30 mL) was combined with distilled water (20 mL), followed by addition of sodium citrate solution (60 mM, 0.46 mL) into a round-bottom flask. This mixture was brought to 90°C and held at this temperature for 5 minutes. Subsequently, $HAuCl_4$ solution (0.23 mL) was introduced in two separate additions, with each addition separated by a 35-minute interval. Upon completion of both $HAuCl_4$ additions, distilled water (20 mL) was incorporated into the reaction mixture. The subsequent growth phase involved adding sodium citrate solution (0.956 mL) to the mixture, which was then heated at 90°C for 5 minutes. Following this heating period, $HAuCl_4$ solution was added in two equal portions (0.478 mL each), maintaining the same 35-minute interval between additions. After both $HAuCl_4$ portions were incorporated, distilled water (27.2 mL) was added to complete the synthesis procedure.

The second synthesis aimed to obtain AuNPs with a diameter of approximately 100 nm. The first step also included seed preparation, however, this time $HAuCl_4$ (25 mM, 0.5 mL) was added to boiling sodium citrate (2.2 mM, 75 mL). The mixture was kept at 90 °C for 30 minutes. To promote the growth of seed particles, fresh stock solutions of $HAuCl_4$ (25 mM) and sodium citrate (60 mM) were prepared. The growth of seed nanoparticles consisted of three stages and the temperature was maintain at 90 °C. In the first growth phase, two portions of $HAuCl_4$ (0.1 mL each) were added to 15.1 mL of the seed solution at 35-minute intervals, followed by the addition of 14.8 mL of water. In the second growth phase, 0.556 mL of sodium citrate solution was added to the reaction mixture. After 5 minutes, two portions of $HAuCl_4$ (0.278 mL each) were introduced at 35-minute intervals. The mixture was then supplemented with 30 mL of water. In the final cycle, 1.133 mL of sodium citrate solution was added to the reaction mixture, followed by the addition of two portions of $HAuCl_4$ (0.567 mL each) at 35-minute intervals, after a 5-minute delay. Finally, the volume was brought up with 36.5 mL of water.

3. Characterization methods

Scanning electron microscopy (SEM) employing a FEI Nova NanoSEM 450 instrument operated at 10 kV was used to analyse the morphology of home-synthesized Au nanoparticles. UV-Vis-NIR spectroscopy employing a Shimadzu 3100 spectrophotometer was used for optical characterization of Au colloidal suspensions, as well as aqueous solutions of Th, Th-CB[7], and Th-CB[8]. Extinction spectra of Au colloids were recorded in PMMA cuvettes (Brand), while absorption spectra of Th, Th-CB[7], and Th-CB[8] were recorded in a quartz cuvette (110-1-40, Hellma).

4. Preparation of plasmonic nanocavities

a. Au oligomers with Th or Th-CB[7]

Aggregation of 48 nm or 96 nm spherical Au nanospheres (as synthesized) was performed by mixing aqueous solution of Th (0.05 mM, 50 μL) or Th-CB[7] solution (aqueous solution of 0.1 mM CB[7] and 0.1 mM Th, 50 μL). Then, the final solution of Au oligomers was drop cast (10 μL) on a piranha-cleaned microscopic cover glass (Brand, No.1) and left to dry under ambient conditions. In case of measurement of solution-phase SERS spectra, aqueous solution of polymer (PVP, 100 μL, 13 wt. %) was added to Au oligomers to stabilize the colloid and prevent further aggregation.[4]

b. Nanoparticle on mirror (NPoM) with Th or Th-CB[7]

Smooth Au substrates were prepared following the procedure proposed by Hegner *et al.*[5] Briefly, 70 nm-thick Au layer was evaporated onto a clean silicon wafer. Then, cover glass (No. 1, Brand) was glued to the gold film using Epo-Tek 377 epoxy glue (both components mixed together in ratio 1:1 by weight) at 150 °C. The glue was cured for 2 h at 150 °C and slowly cooled down, after which it was pulled off to reveal flat Au substrates. Au substrate were factionalized by submersion in the host–guest solution (mixed aqueous solution of 1 mM CB[7] and 0.1 mM Th (or 0.01 mM for single-molecule detection)) for at least 24 h. In case of sample without CB[7] Au substrate was submerged in the aqueous solution of Th (5 µM or 0.05 µM for single-molecule detection). Substrates were then rinsed with water and blow-dried with nitrogen. Afterwards, 48 nm or 96 nm Au nanoparticles (Figure S1) were drop cast onto Au substrate. After around 5 min, excess of Au nanoparticles were washed away and substrate was blow-dried with nitrogen.

5. Raman and SERS measurement

Raman and SERS measurements were performed using an InVia Renishaw Raman microspectrometer equipped with Leica microscope and a thermoelectrically cooled 1021 × 256 pixels CCD detector. The Raman spectra of crystals and SERS spectra of dry samples were collected using a 50× Zeiss objective (NA = 0.95), while liquid samples were collected in 10 mm cuvette (104-002-10-40, Hellma) using a 30 mm lens in a back-scattering configuration. The signal was dispersed by 1200 or 1800 grooves per mm holographic gratings. Three laser lines were used for excitation: 532 nm (CNI, Optoelectronics Tech. Co., Ltd. MSL-S-532B), 633 nm (HeNe, Renishaw), and 785 nm (HPNIR785). Raman and SERS mapping were carried out by raster scanning the sample in the horizontal (XY) plane and registering Raman spectra at each point. Renishaw Wire 5.6 software was used to obtain the intensity distribution map and remove contributions from cosmic rays.

A part of SERS measurements of single-molecule trajectories of Th-CB[7] were performed on a home-built SERS microscopy set-up, described in detail elsewhere.[6] However, all measurement parameters and conditions, including excitation wavelength, exposure time, power on the sample, and objective, remained identical to those during measurements with the commercial inVia Renishaw spectrometer.

6. Numerical simulations

Simulations of the electric-field distribution in the plasmonic nanocavities were performed with Asnys Lumerical FDTD. Nanocavities were modelled as Au nanosphere on top of thick Au layer in order to simulate the experimental NPoM geometry, or as a dimer of two Au nanosphere to simulate the experimental oligomers geometry. The gap between Au nanoparticle and Au layer / Au nanoparticle was set to 0.9 nm. In the region of gap the mesh was set to 0.1 nm in all directions, and outside the gap region was set to 1 nm. The dielectric function of Au was taken from Johnson and Christy.[7] To account for the experimental sample illumination via a high-numerical aperture objective, the FDTD simulations were performed with a p-polarized plane wave (TFSF source) at two angles of incidence: $\theta = 0°$ and $\theta = 71.28°$.

7. Quantum-chemical calculations

For molecular simulations, the CB[7] starting geometry was obtained from the Cambridge Structural database[8] (cucurbit[7]uril, refcode FUYHIR). In case of thionine molecule and CB[7]-Th complex starting geometry were built and optimized with Gabedit software. Calculations were made using PBE1PBE/Def2TZV functional and basis set combination using Gaussian 16. Geometries were first optimized and then vibrational analysis was performed. Finding of stationary point was confirmed by the absence of imaginary vibrations. Raman intensity spectra have been calculated from Gaussian output files Raman activities using Chemcraft visualization software.[9]

The visualization of Raman tensors of selected vibrational modes for Th before and after encapsulation inside CB[7] were performed using home-written Matlab code based on procedure described in ref.[10]

II. Additional results

1. Characterization of Au nanoparticles

Representative SEM micrographs, as well as extinction spectra of the prepared Au nanoparticles are presented in Figure S1(B), Figure S1(D), and Figure S1(A) . Morphological analysis of individual spherical particles from SEM imaging revealed average diameters of 48 ± 10 nm (Figure S1(C)) and 97 ± 2 nm (Figure S1(E)).

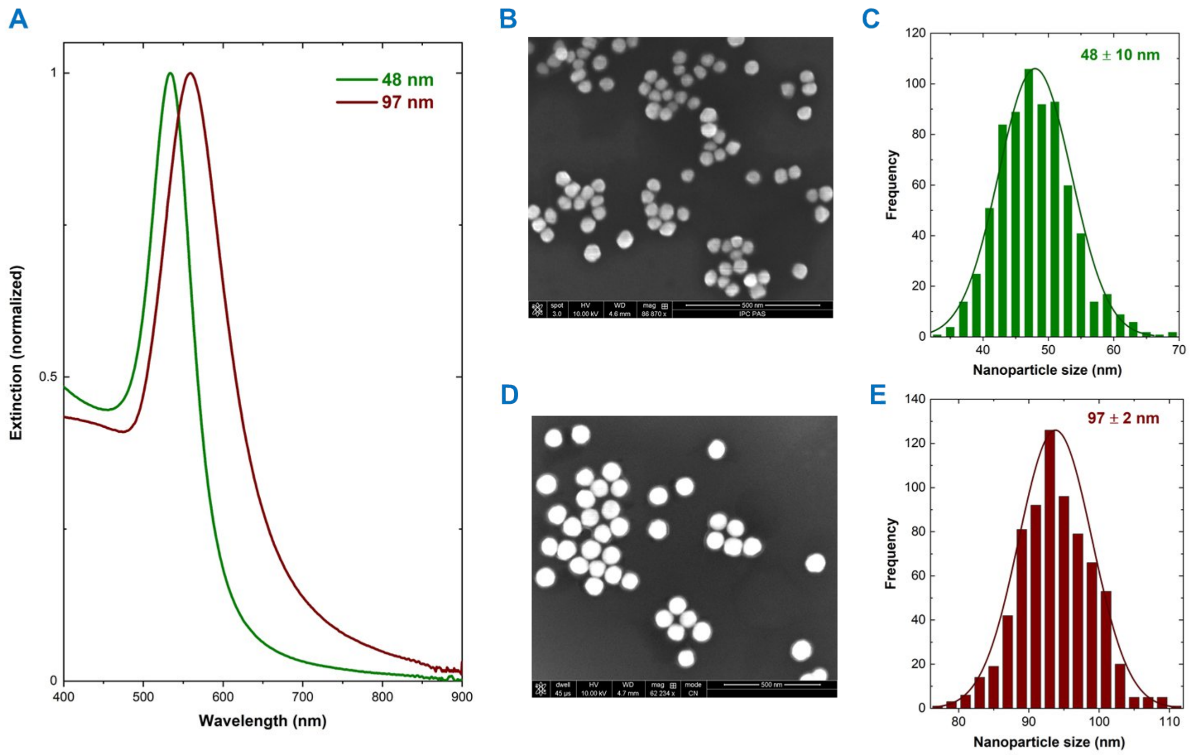

Figure S1. Extinction spectra of Au colloids (A). SEM images of Au nanoparticles drop casted on silicon substrate (B and D). Nanoparticle diameter distribution (C and E).

2. Encapsulation of thionine in cucurbit[7]uril

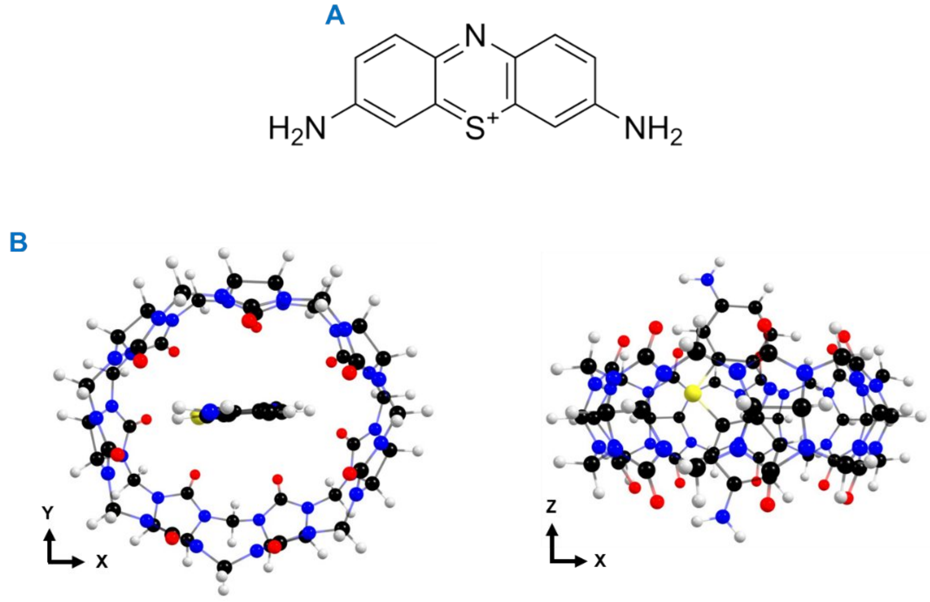

Figure S2. Chemical structure of Th. (A) Top (left) and side (right) view on Th-CB[7] structure optimized with DFT. (B)

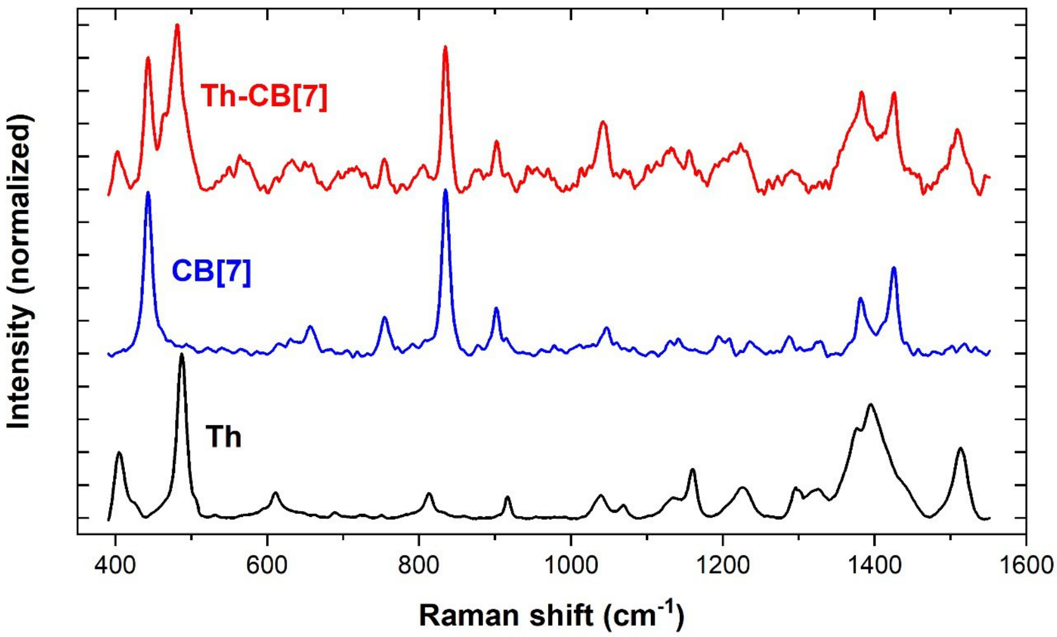

Figure S3. Room temperature bulk Raman spectra of a aqueous solution of Th (~0.9 mM) (**black**), CB[7] (~25 mM) (**blue**), and Th-CB[7] (**red**) exited at 785 nm (accumulation time = 90s, 30 accumulations, power on sample 28.6 mW). The spectra are baseline corrected and normalized for clarity.

3. Raman and SERS characteristics of thionine with varying excitation wavelengths

a. Raman and SERS spectra of thionine

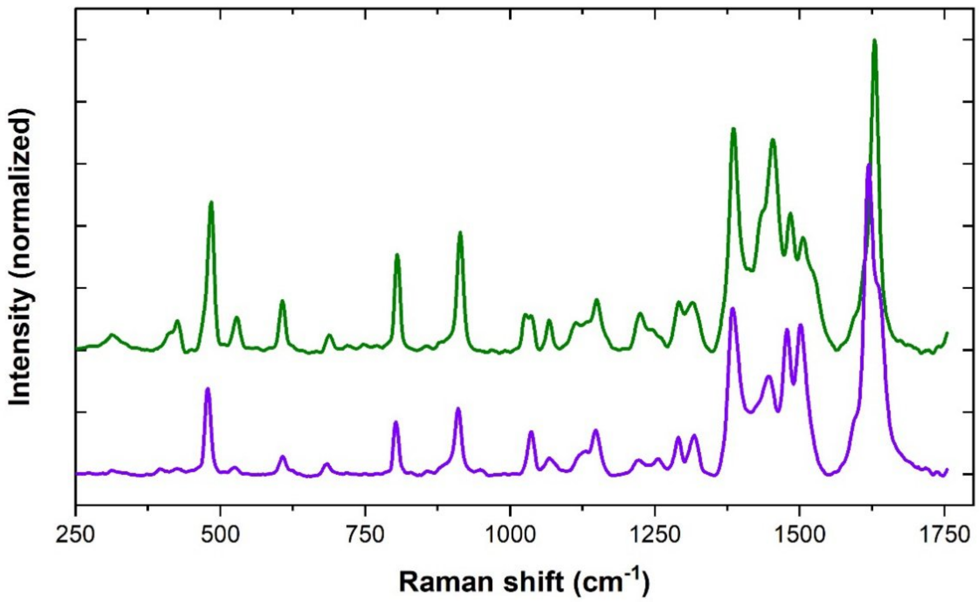

Figure S4. Room temperature Raman spectra of a crystalline Th (**green**) and averaged SERS spectrum of Th on Au spherical oligomers (**purple**) exited at 532 nm.

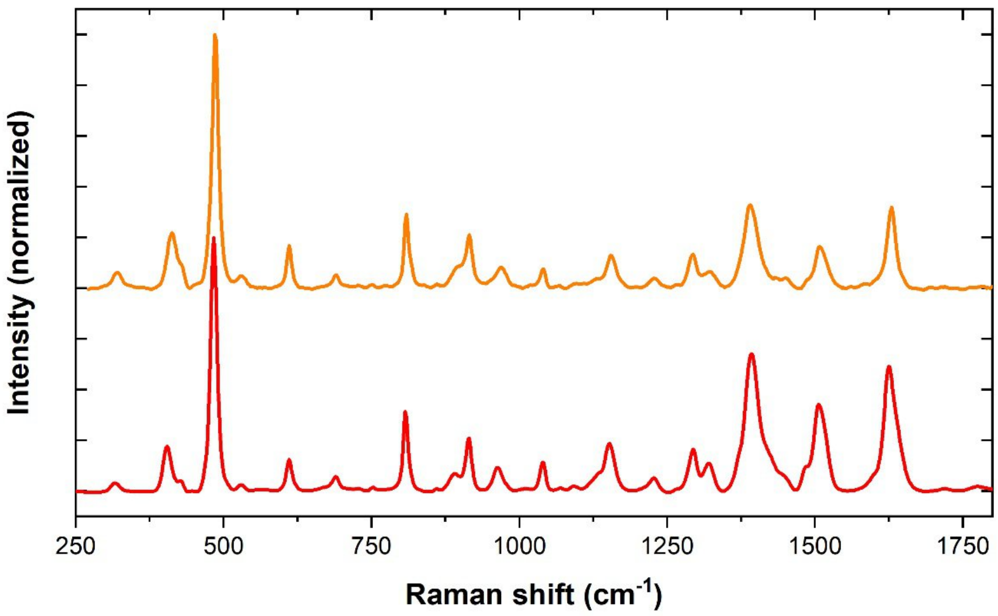

Figure S5. Room temperature Raman spectra of a crystalline Th (**orange**) and averaged SERS spectrum of Th on Au spherical oligomers (**red**) exited at 633 nm.

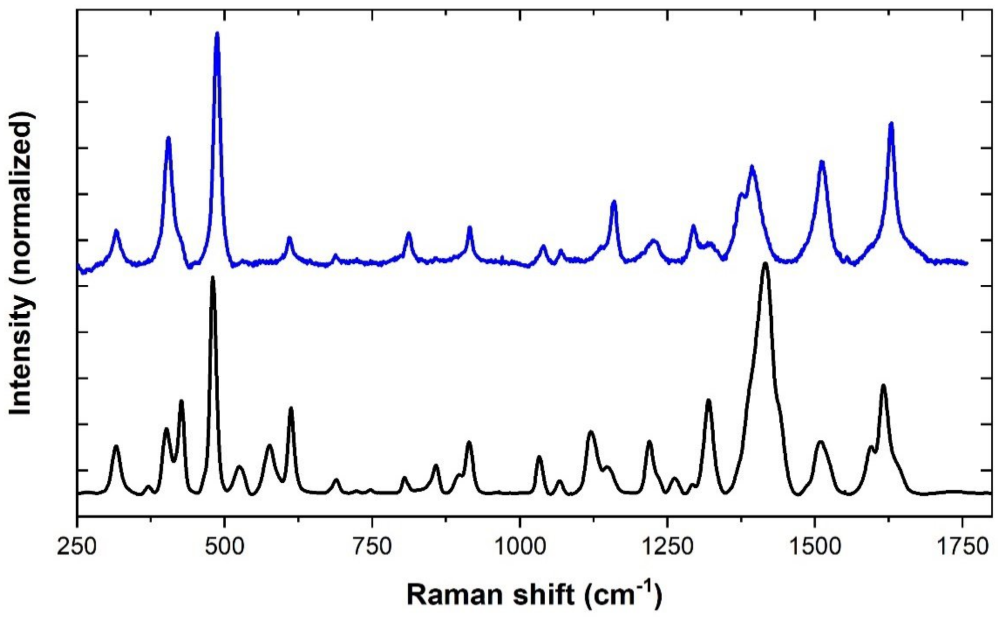

Figure S6. Room temperature Raman spectra of a aqueous solution of Th (**blue**) and averaged SERS spectrum of Th on Au spherical oligomers (**black**) exited at 785 nm.

b. Raman and SERS bands assignments

The assignment of Th bands was made based on the measurements of spontaneous Raman and SERS spectra taking into account different forms of the studied molecule and variable excit-

ation wavelengths, as well as literature[11–14]. The experimental spectra were compared with simulated one (PBE1PBE/Def2TZV).

Table S1. Peak assignment of thionine (Th) based on experimental data, quantum-chemical simulations, and literature.

Observed					Calculated	Peak assigment	Ref.
Raman		SERS					
crystal (532 nm)	water (785 nm)	532 nm	633 nm	785 nm	wavenumber (cm^{-1})		
312	**316**	312	316	**316**	310	bending vibration resulting from ν(C–S–C)	13
		373		**371**	358		
411	**405**	395	404	**401**	401		
425		424	427	**427**	427		
484	**487**	478	483	**480**	478	skeletal deformation resulting from ν(C–N–C)	11
528		523	530	**525**	548		
				**577**			
608	**609**	607	610	**612**	609	skeletal deformation resulting from ν(C–S–C)	11,13
688	**687**	684	689	**689**	703		
805	**809**	803	807	**805**	815	Asymmetric skeletal deformation resulting from ν(C–N–C), ν(C–S–C), and ν(C–C–C)	13
		857	860	**858**	845		
	**893**		890	**897**	883		
914	**915**	912	913	**913**	923		
			963	**964**	995		
1027							
1036	**1039**	1036	1039	**1033**	1052	in-plane bending of C–H	11,14
1067	**1070**	1068	1070	**1067**	1083		
			1092				
		1127	1132	**1121**			
1147	**1159**	1147	1151	**1150**	1195		
1224	**1228**	1221	1227	**1219**	1277		
		1255		**1262**			
1295	**1294**	1290	1293	**1292**			
1316	**1324**	1317	1320	**1319**	1324	Ar–N stretching	11
1374	**1374**			**1371**	1379		
1385	**1394**	1384	1392	**1390**	1409		
1411			1420	**1415**	1420	asymmetric stretching vibration of C–N and ν_{asym}(C–N–C)	14
1434		1446	1445	**1440**	1448		
1483		1478	1484	**1485**	1523		
1507	**1512**	1501	1508	**1512**	1530	asymmetric skeletal deformation of ν(C–C–C) and ν(C–C)	11, 13
1524					1570		
1593				**1595**	1669	ν(C–C) ring stretching vibration	11, 12
1630	**1629**	1619	1626	**1616**	1694	ν(C–C) ring stretching vibration	11, 12
		1636		**1640**	1718		

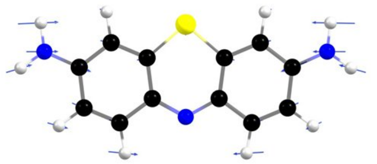
mode 310 cm^{-1}

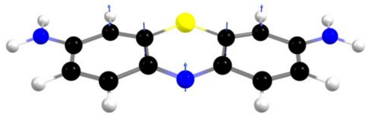
mode 358 cm^{-1}

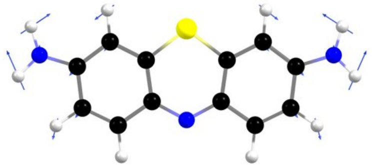
mode 401 cm^{-1}

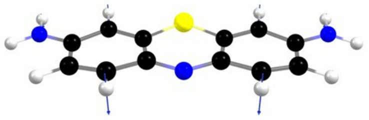
mode 427 cm^{-1}

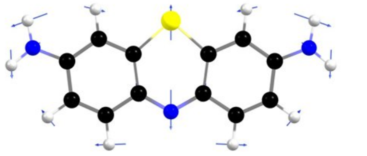
mode 478 cm^{-1}

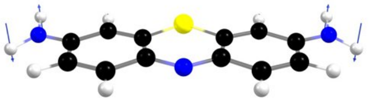
mode 548 cm^{-1}

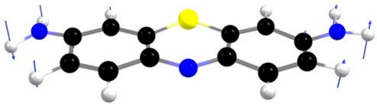
mode 609 cm^{-1}

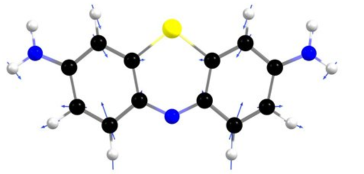
mode 703 cm^{-1}

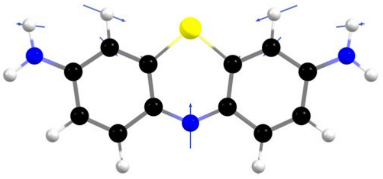
mode 815 cm^{-1}

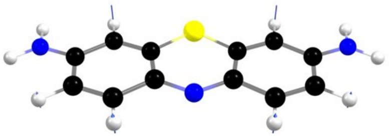
mode 845 cm^{-1}

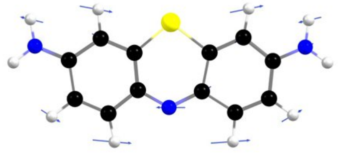
mode 883 cm^{-1}

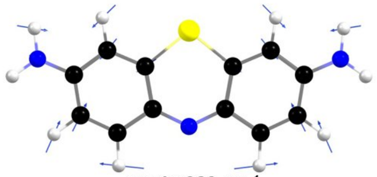
mode 923 cm^{-1}

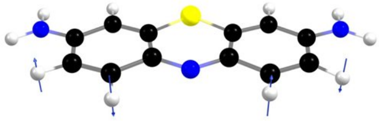
mode 995 cm^{-1}

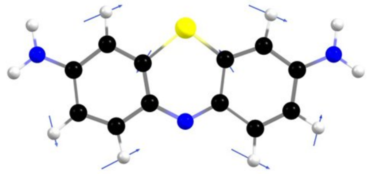
mode 1052 cm^{-1}

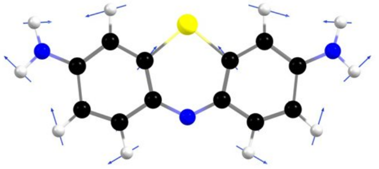
mode 1083 cm^{-1}

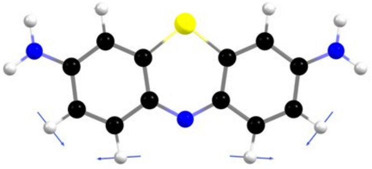
mode 1195 cm^{-1}

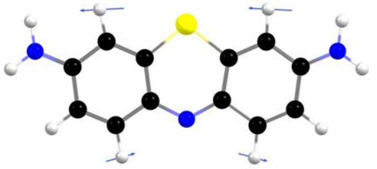
mode 1277 cm^{-1}

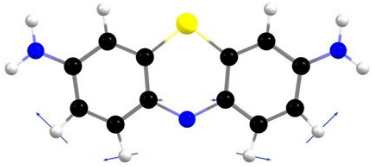
mode 1324 cm^{-1}

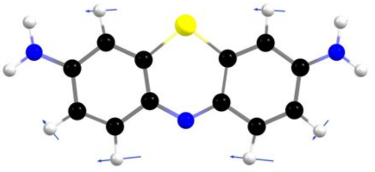
mode 1379 cm^{-1}

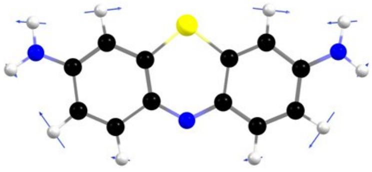
mode 1409 cm^{-1}

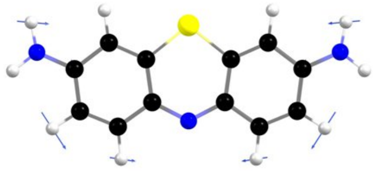
mode 1420 cm^{-1}

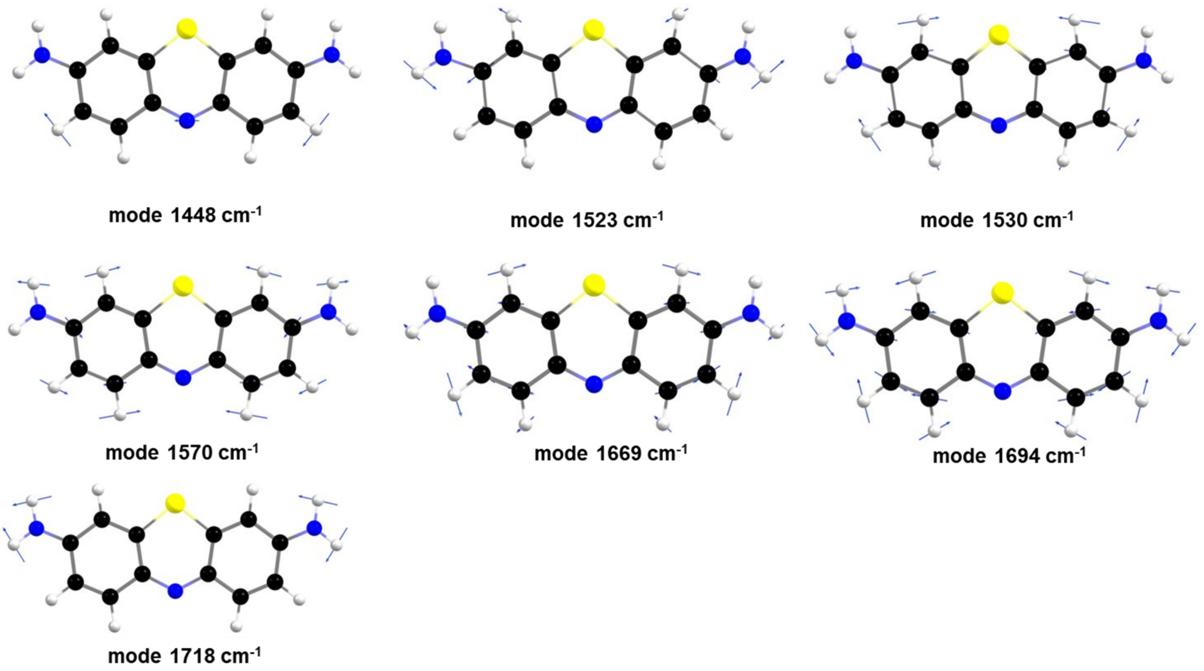

Figure S7. Selected vectors of the normal vibrational modes of Th and their frequencies, calculated using the PBE1PBE/Def2TZV method.

4. Pre-resonance (excitation wavelength 633 nm) SERS of Th and Th-CB[7] in a dry and wet environments

Here, we present SERS spectra recorded using a 633 nm excitation wavelength. Each spectrum represents an average, with the standard deviation shown as a shaded area alongside the corresponding spectrum. For the "wet" sample, the SERS spectra were measured with a laser power of 0.2 mW, whereas for the "dry" sample the power was 0.77 mW. In both cases, the accumulation time was set to 1 s. Because the SERS spectra of the "wet" sample are much more reproducible, the corresponding standard deviations are very small and therefore not visible in Fig. S8A.

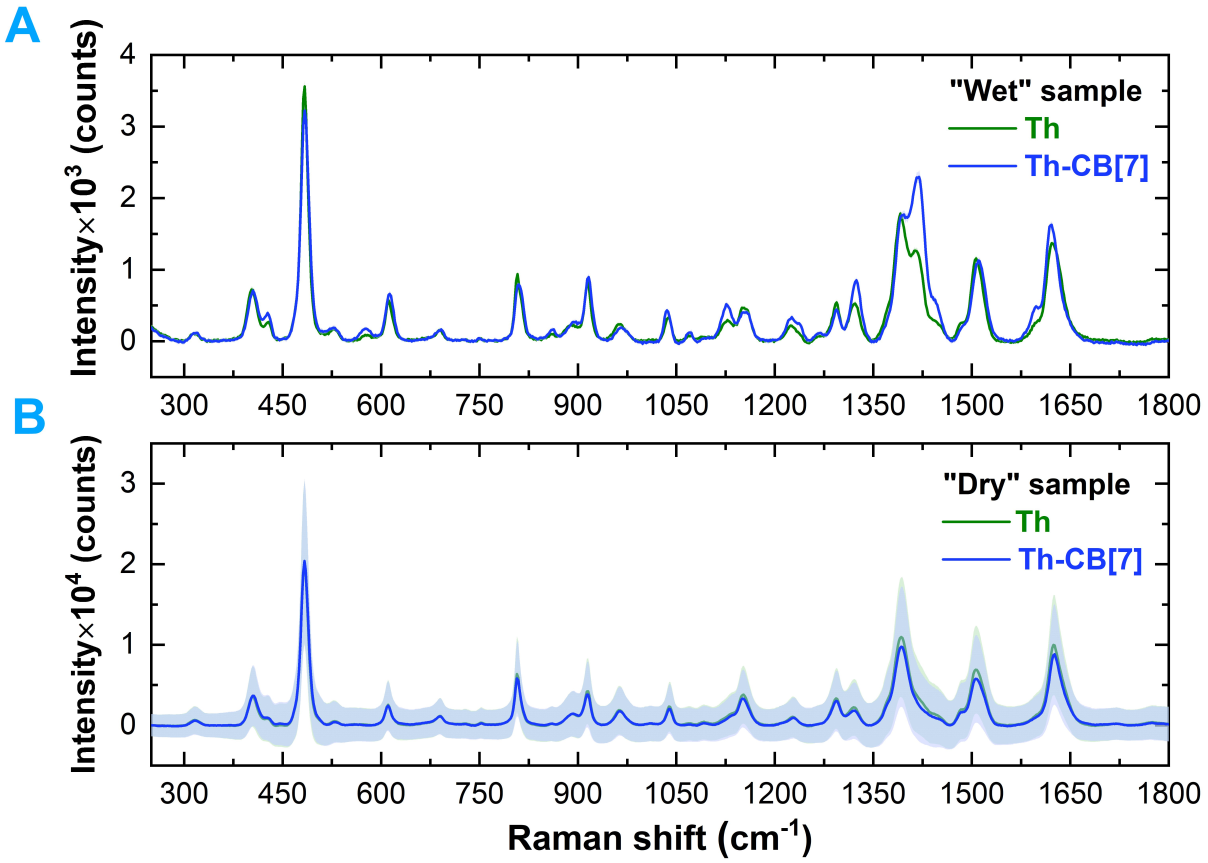

Figure S8. Baseline-corrected average SERS spectra of Th and Th-CB[7] registered with excitation 633 nm on colloidal solution of spherical Au oligomers (A) and deposited dry spherical Au oligomers (B).

5. SERS signal decay profiles for Th and Th-CB[7] – kinetic parameters

Here we present the values of kinetic parameters for the SERS signal decay profiles of Th or Th-CB[7] shown in Figure 4 in the main manuscript.

Table S2. Exponential-decay fitting parameters: τ_1, τ_2 — time constant; SE — standard error; RSS — residual sum of squares; RMSE — root-mean-square error.

Excitation in pre-resonance (633 nm)							
		τ_1 (s)	SE τ_1	τ_2 (s)	SE τ_2	RSS	RMSE
**NPoM with Th**	mono	8.9	0.47			0.0045	0.0098
**NPoM with Th-CB[7]**	mono	6.2	0.57			0.0470	0.0316
**Au oligomers with Th**	mono	4.5	0.28			0.0314	0.0258
	bi	1.3	0.10	12.6	1.52	0.0028	0.0078
**Au oligomers with Th-CB[7]**	mono	3.3	0.18			0.0476	0.0318
	bi	0.8	0.04	6.5	0.26	0.0021	0.0068
**Excitation in of-resonance (785 nm)**							
**NPoM with Th**	mono	12.0	1.14			0.0150	0.0179
**NPoM with Th-CB[7]**	mono	4.6	0.32			0.0314	0.0258
**Au oligomers with Th**	mono	6.1	0.32			0.0281	0.0245
	bi	1.5	0.15	13.3	1.52	0.0036	0.0090
**Au oligomers with Th-CB[7]**	mono	7.1	0.11			0.0030	0.0081
	bi	5.3	1.19	21.7	35.06	0.0018	0.0063

6. Numerical simulations

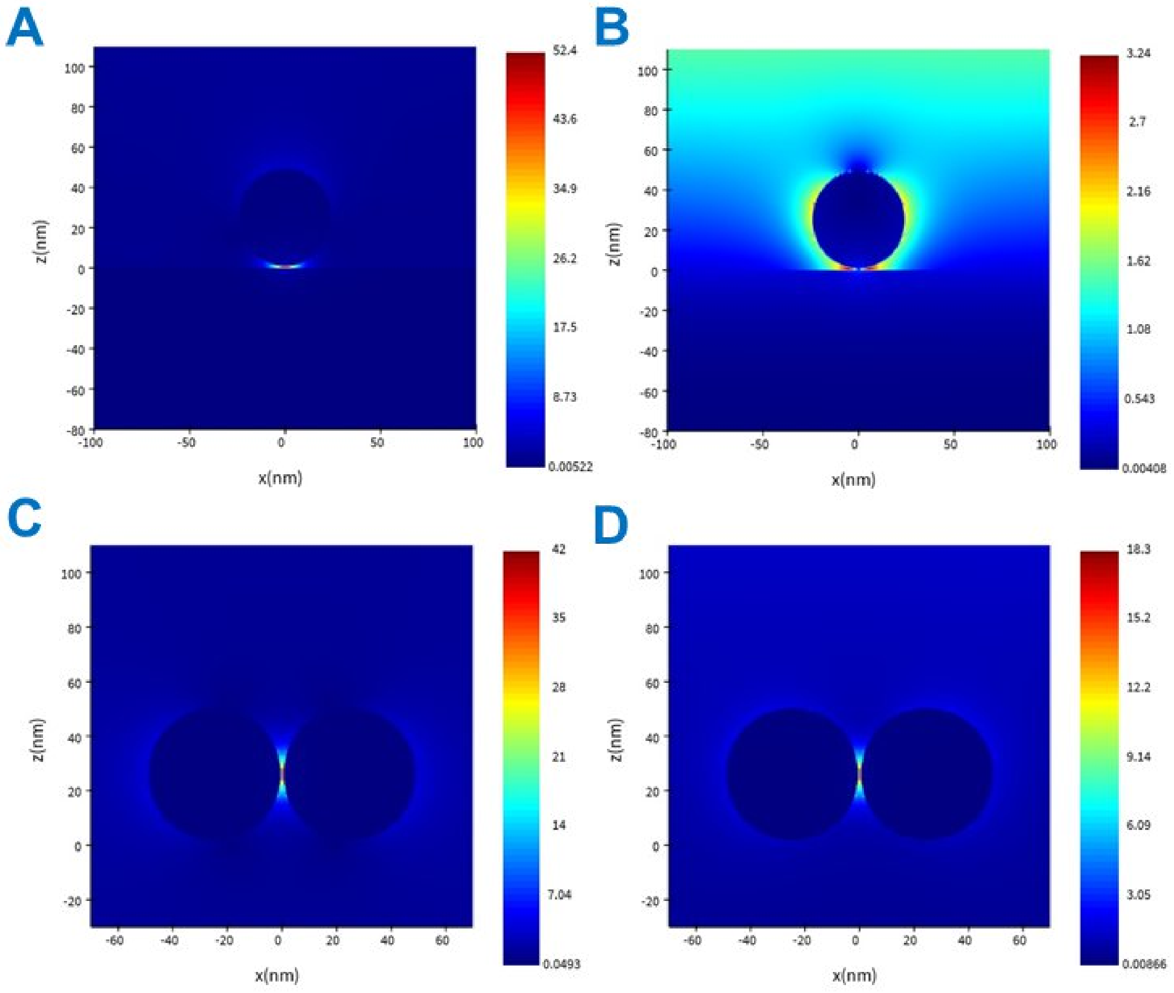

Figure S9. FDTD calculated electric field enhancement distribution in nanocavities composed of nanoparticles with a diameter of 48 nm. NPoM illuminated at 71.28° (A) and 0° (B). Au nanosphere dimer illuminated at 0° (C) and 71.28° (D).

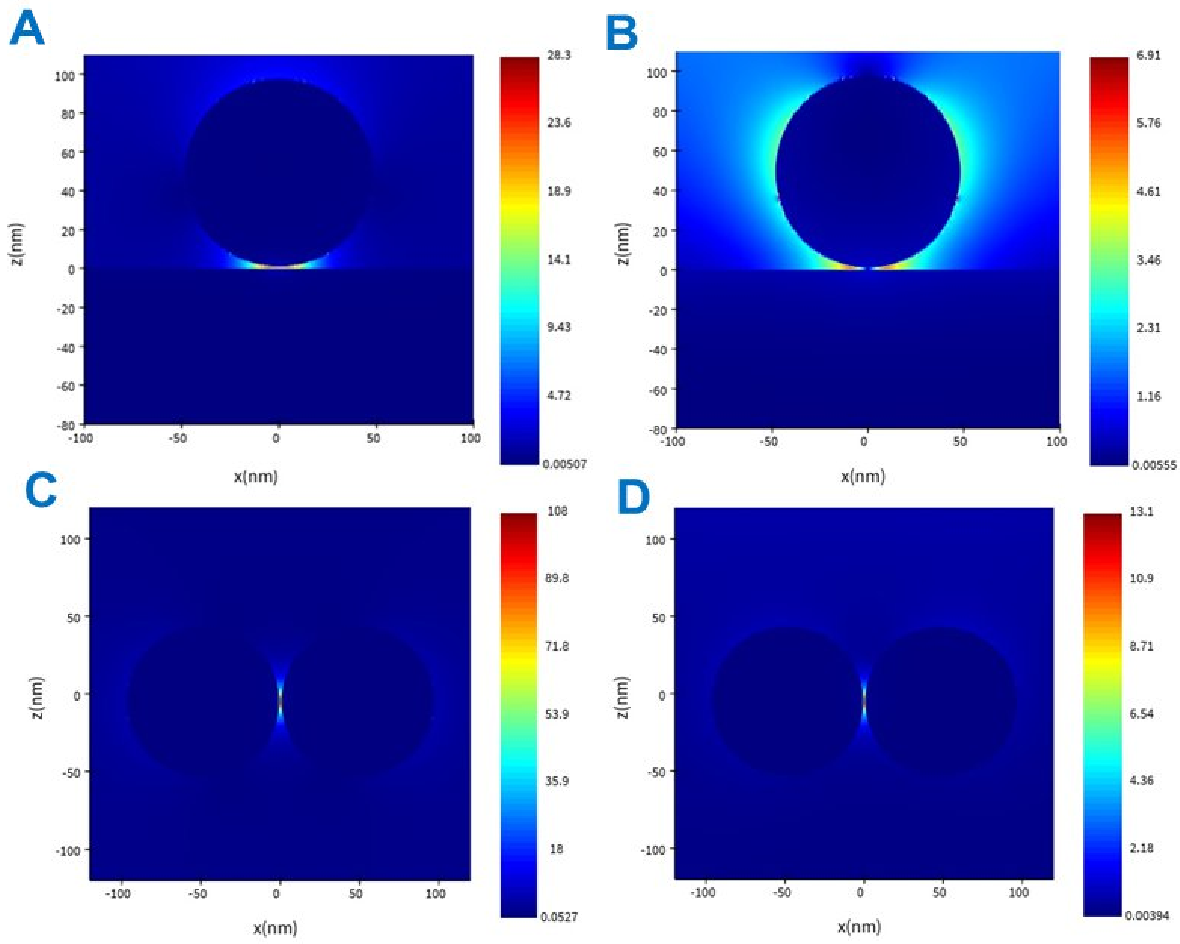

Figure S10. FDTD calculated electric field enhancement distribution in nanocavities composed of nanoparticles with a diameter of 97 nm. NPoM illuminated at 71.28° (A) and 0° (B). Au nanosphere dimer illuminated at 0° (C) and 71.28° (D).

7. **Temporal evolution of SM-SERS spectra for Th and Th-CB[7] recorded in the NPoM cavity**

Here, we present additional SERS trajectories of single Th (A) or single Th-CB[7] (B) registered from different NPoM hotspots.

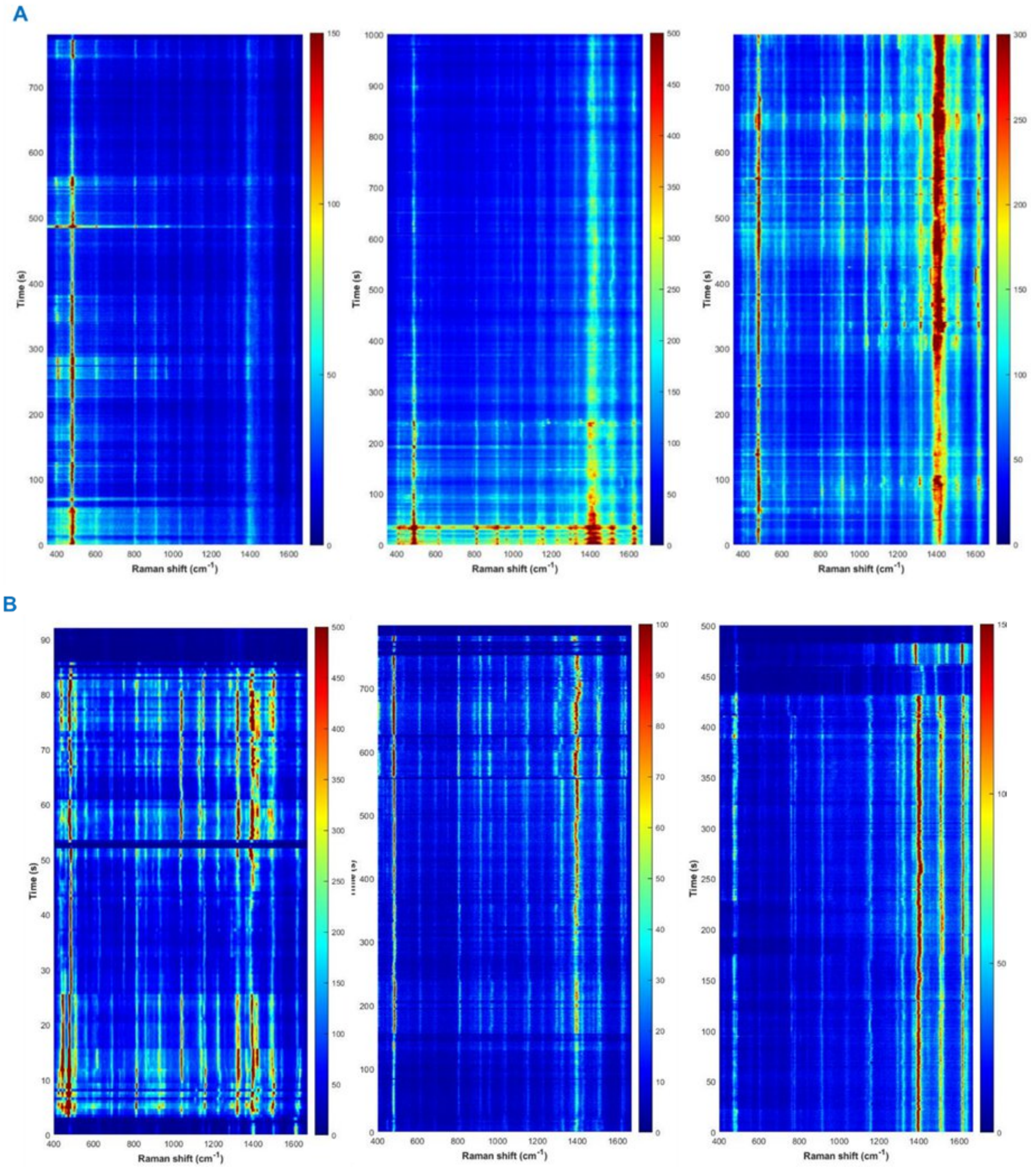

Figure S11. Time dependent evolution of SERS signal taken from NPoM nanocavity with Th molecules (A), or Th-CB[7] (B). Each spectrum is collected for 0.5 s with 633 nm excitation and 17 μW laser power on the sample.

III. References

1. N. G. Bastús, J. Comenge and V. Puntes, *Langmuir*, 2011, **27**, 11098–11105.
2. J. H. Yoon, J. Lim and S. Yoon, *ACS Nano*, 2012, **6**, 7199–7208.
3. J. Turkevich, P. C. Stevenson and J. Hillier, *Discuss. Faraday Soc.*, 1951, **11**, 55–75.
4. B. Negru, M. O. Mcanally, H. E. Mayhew, T. W. Ueltschi, L. Peng, E. A. Sprague-Klein, G. C. Schatz and R. P. Van Duyne, *J. Phys. Chem. C*, 2017, **121**, 27004–27008.
5. M. Hegner, P. Wagner and G. Semenza, *Surf. Sci.*, 1993, **291**, 39–46.
6. A. Amirsalari, S. Razi and S. Gawinkowski, *J. Raman Spectrosc.*, 2023, **54**, 976–987.
7. P. B. Johnson and R. W. Christy, *Phys. Rev. B*, 1972, **6**, 4370–4379.
8. F. H. Allen, *Acta Crystallogr. Sect. B Struct. Sci. Cryst. Eng. Mater.*, 2002, **58**, 380–388.
9. P. L. Polavarapu, *J. Phys. Chem.*, 1990, **94**, 8106–8112.
10. E. C. Le Ru and P. G. Etchegoin, *Principles of Surface-Enhanced Raman Spectroscopy*, Elsevier Science, 2009.
11. C. Ruan, W. Wang and B. Gu, *J. Raman Spectrosc.*, 2007, **38**, 568–573.
12. K. Hutchinson and E. Hester, *J. Chem. Soc. Faraday Trans.*, 1984, **80**, 2053–2071.
13. W. Xu, M. Aydin, S. Zakia and D. L. Akins, *J. Phys. Chem. B*, 2004, **108**, 5588–5593.
14. H. R. Virdee and R. E. Hester, *Laser Chem.*, 1988, **9**, 401–416.
